# Hydrogel Microspheres for Biomedical Applications

**DOI:** 10.1002/smsc.202500453

**Published:** 2025-11-18

**Authors:** Yingkang Huang, Xi Zhu, Jinhong Zhou, Henan Li, Wei Zhang, Danyang Shen, Ziyan Huang, Tianbo Zhang, Lin Zhuang, Lei Qin, Xiaofeng Xue, Yunjie Lu

**Affiliations:** ^1^ Department of General Surgery The First Affiliated Hospital of Soochow University Suzhou 215006 China; ^2^ Department of Infection Kunshan First People's Hospital Kunshan Jiangsu 215300 China; ^3^ Department of General Surgery Taizhou Second People's Hospital Affiliated to Yangzhou University Taizhou Jiangsu 225300 China; ^4^ Stanford Cardiovascular Institute Stanford University School of Medicine Stanford CA 94305 USA; ^5^ Department of General Surgery Wujin Affiliated Hospital of Jiangsu University and The Wujin clinical college of Xuzhou medical university Changzhou Jiangsu 213100 China

**Keywords:** biobased materials, biomedical applications, drug delivery, hydrogel microspheres, preparation methods

## Abstract

With the advancement of hydrogel technology, increasing attention has been drawn to hydrogel microspheres (HMs) due to their versatile biomedical applications. HMs play pivotal roles in biomedical applications, such as drug delivery, cell culture, regenerative medicine, wound healing, and tumor immunity. Composed of diverse biobased materials and fabricated through various preparation methods, HMs offer unique structural and functional advantages. This review focuses on the latest findings to provide a more comprehensive understanding of HMs for biomedical applications. Their therapeutic potential across multiple disease contexts is highlighted, and emerging trends and challenges are discussed. By consolidating current knowledge, this work aims to inspire further research and accelerate the clinical translation of HMs.

## Graphical abstract

Hydrogel microspheres (HMs) play pivotal roles in biomedical applications, such as drug delivery, cell culture, regenerative medicine, wound healing, and tumor immunity. This review focuses on the latest findings to provide a more comprehensive understanding of HMs for biomedical applications. Their therapeutic potential across multiple disease contexts is highlighted, and emerging trends and challenges are discussed.

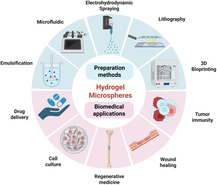

## 1 Introduction

Over the past few decades, research on hydrogel molding technology has advanced rapidly, leading to its widespread application in the biomedical field. Hydrogels are a class of 3D polymeric networks composed of hydrophilic polymers that can absorb and retain large amounts of water while maintaining their structural integrity and soft, tissue‐like properties.^[^ ^1^, ^2^ ^]^ For instance, dual‐crosslinked hydrogels not only mimic the structural features of the growth plate but also enhance local expression of IGF‐1, thereby effectively modulating the functional behavior of growth plate tissue.^[^ ^3^ ^]^ In addition, hydrogels have shown promising applications in the management of dental caries, leveraging their antibacterial activity and potential to promote remineralization.^[^ ^4^ ^]^ Conventional bulk hydrogels, although effective in facilitating controlled drug release and supporting tissue engineering applications, possess several limitations.^[^ ^5^, ^6^ ^]^ Their millimeter‐scale dimensions often necessitate invasive implantation, impede efficient nutrient diffusion, and result in uneven cell distribution.^[^ ^7^ ^]^ To overcome these challenges, micro‐ and nanoscale hydrogel structures, such as microspheres, have emerged as promising alternatives.^[^ ^8^ ^]^ These microspheres exhibit notable advantages such as high specific surface area, tunable mechanical properties, and considerable design flexibility, rendering them ideal building blocks for engineering specific biological functions.

Hydrogel microspheres (HMs) are micron‐sized spherical structures formed through the physical or chemical crosslinking of hydrophilic or amphiphilic polymers.^[^ ^9^, ^10^ ^]^ Their porous architecture closely mimics the natural extracellular matrix (ECM), allowing them to simulate the in vivo microenvironment and respond dynamically to physiological stimuli.^[^ ^11^ ^]^ Compared to conventional bulk hydrogels, HMs offer several core advantages. They enable minimally invasive delivery to target tissues via syringes or catheters, thereby avoiding surgical trauma. Owing to their injectability and capacity for multifunctional modification, HMs can effectively modulate pathophysiological processes in cells and the ECM at cartilage lesion sites.^[^ ^12^ ^]^ Furthermore, HMs support modular design strategies. The combination of microspheres with distinct functions (e.g., drug‐loaded, cell‐laden, or imaging‐enabled) allows the construction of heterogeneous materials to address complex therapeutic requirements.^[^ ^13^ ^]^ More importantly, the incorporation of stimuli‐responsive groups (e.g., to light, heat, pH, or glucose) confers dynamic responsiveness upon HMs, facilitating precise spatiotemporal drug release.^[^ ^14^, ^15^ ^]^

Several hydrogel‐based therapies have already made significant clinical impacts. For instance, Seprafilm, an adhesion barrier composed of hydrogel, has received FDA approval for minimizing postsurgical tissue adhesions.^[^ ^16^ ^]^ Synvisc, a hyaluronic acid hydrogel, is widely used as a viscosupplement for osteoarthritis treatment.^[^ ^17^, ^18^ ^]^ Additionally, Regranex, a hydrogel incorporating platelet‐derived growth factor, has been clinically approved to enhance the healing of diabetic foot ulcers.^[^ ^19^ ^]^ These established clinical applications underscore the significant translational potential of hydrogel technology and thereby motivate continued research into HMs as next‐generation biomaterials poised to address more complex biomedical challenges. In addition to conventional hydrogel‐based therapies, HMs have developed as a unique and highly adaptable platform, demonstrating considerable potential for clinical applications. Their diminutive dimensions and capacity for injection facilitate minimally invasive administration and targeted access to localized treatment areas.^[^ ^20^, ^21^ ^]^ A prominent example is their use as embolic agents in transarterial chemoembolization (TACE), where drug‐eluting HMs (such as LC Bead) are deployed to block tumor‐feeding arteries while simultaneously providing localized, sustained chemotherapy release, markedly improving outcomes for hepatocellular carcinoma patients.^[^ ^22^ ^]^ Furthermore, BST‐CarGel, a cartilage repair scaffold composed of HMs, has progressed to the clinical trial phase. It functions by offering a 3D scaffold structure and enabling the sustained release of growth factors, thereby facilitating minimally invasive repair of articular cartilage defects.^[^ ^23^ ^]^ Moreover, HMs are extensively investigated as controlled drug delivery systems for a wide range of bioactive molecules, including peptides, antibodies, and vaccines, owing to their tunable degradation and release kinetics. Despite these advancements, significant challenges persist in the precise regulation of their mechanical characteristics, degradation behaviors, and multifunctional integration for advanced applications.

**Figure 1:**
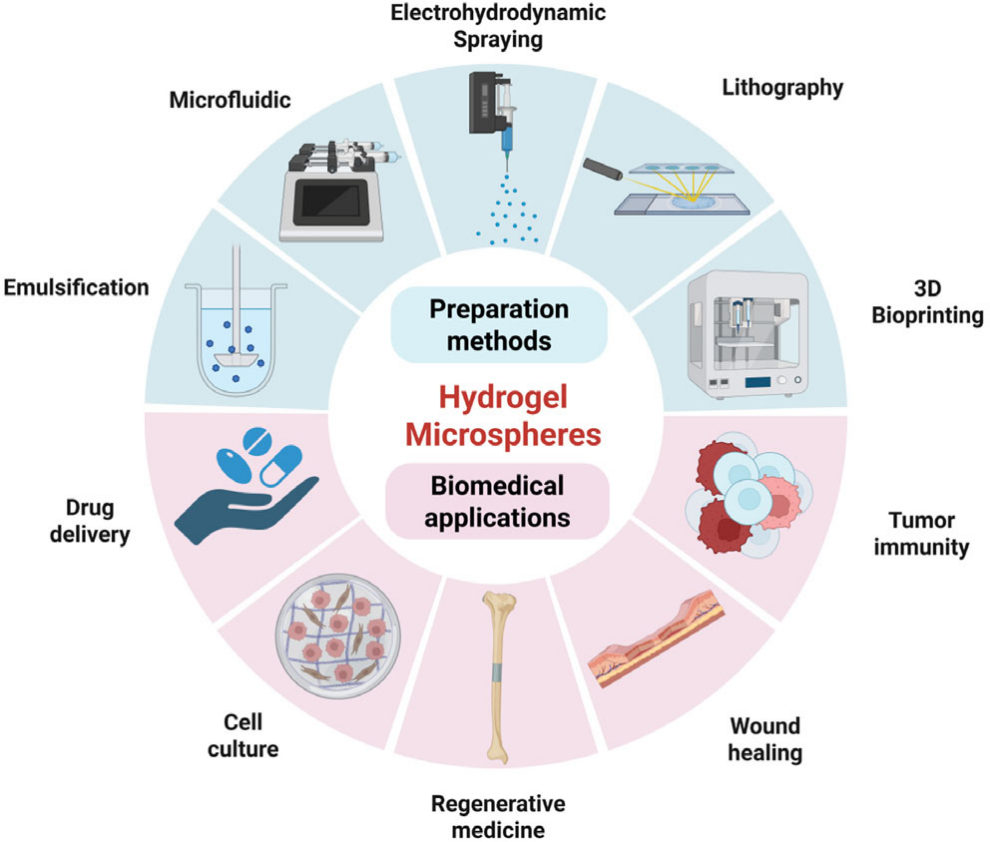
Preparation methods and biomedical applicationsof HMs. The figure was created with BioRender.

This review charts the progress of HMs by systematically summarizing recent advances and emerging trends in biomedical applications. We critically analyze the foundational materials and fabrication techniques, with dedicated focus on elucidating innovative applications and their functional mechanisms across key areas, including drug delivery, cell culture, regenerative medicine, wound healing, and tumor immunity (**Figure** **1**). Concurrently, we will address the existing bottlenecks and challenges prevalent in current research, offering a forward‐looking perspective on the future developmental trajectory of this dynamic field. Through this review, we aspire to furnish researchers with a comprehensive knowledge repository, inspire innovative thought, and collaboratively enhance the significant impact of HM technology on human health.

## 2 Biobased Materials

### 2.1 Natural Materials

Natural polymers are derived directly from botanical, animal, or human sources and are characterized by favorable properties including high biocompatibility, low toxicity, and minimal immunogenicity. In this section, we survey a range of natural polymers employed in the fabrication of HMs, covering alginate, chitosan, collagen, gelatin, hyaluronic acid, and cellulose, as well as other natural materials such as chondroitin sulfate, agarose, fibrin, and silk fibroin.

#### 2.1.1 Alginate

Alginate is a naturally derived polysaccharide extensively employed in the preparation of HMs, a preference attributed to its favorable properties, including remarkable biocompatibility and biodegradability. Sourced from brown seaweeds, its molecular structure consists of α‐D‐mannuronic acid and β‐L‐guluronic acid.^[^ ^24^ ^]^ In addition, alginate hydrogels can be quickly crosslinked using divalent metal conditions such as calcium (Ca^2+^) at mild pH and temperature levels, which may be helpful to maintain the bioactivity of the drugs and cells.^[^ ^25^ ^]^

The alginate hydrogel network forms a protective barrier that shields encapsulated active substances from degradation in the gastric environment, improving their stability and bioavailability. For instance, alginate microspheres loaded with M2 macrophage membrane‐coated Janus nanomotors have been developed for targeted ulcerative colitis therapy.^[^ ^26^ ^]^ After oral administration, it is not affected by the harsh gastric environment and is released in the intestine. There was a notable reduction in the severity of ulcerative colitis, which encompassed a decrease in inflammation, the neutralization of reactive oxygen species (ROS), the reprogramming of macrophages, and the restoration of both the intestinal barrier and microbiota.^[^ ^26^, ^27^ ^]^ The incorporation of halloysite clay nanotubes and metal–polyphenol networks further enhances this system by prolonging probiotic survival and sealing hydrogel pores against harmful substances.^[^ ^28^ ^]^ The mechanical strength and stability of the microspheres can be precisely regulated by adjusting the concentrations of alginate and crosslinking agent, along with the duration of the crosslinking process. At the same time, the carboxyl (–COOH) and hydroxyl (–OH) on the alginate chain provide convenient chemical modification sites that can be grafted onto other polymers, targeted ligands, or functional molecules to improve the performance of microspheres. Nonetheless, the HMs synthesized from natural alginate crosslinked with metal cations exhibit a slow degradation rate and possess suboptimal mechanical properties. The incorporation of xonotlite nanowires as bioactive enhancers addressed the mechanical constraints of alginate microspheres, leading to a marked increase in the expression of osteogenic genes within encapsulated BMSCs and effectively promoting bone regeneration in the rat femoral bone defect model.^[^ ^29^ ^]^ Another limitation of pure alginate is its lack of inherent cell adhesion motifs, which restricts cellular adhesion and proliferation. To confer bioadhesive properties, alginate HMs can be functionalized with arginine–glycine–aspartic acid (RGD) peptides or laminin. Such RGD‐modified alginate microspheres have demonstrated the ability to support the adhesion and proliferation of human umbilical vein endothelial cells (HUVECs), highlighting their potential as cell delivery carriers in regenerative medicine and tissue engineering.^[^ ^30^ ^]^

With its excellent biocompatibility, controllable mechanical properties, and outstanding gelation capacity, alginate presents itself as an ideal foundational material for fabricating HMs. Through reasonable preparation methods and modification strategies, trehalose HMs have demonstrated broad application prospects in fields such as tissue engineering, drug delivery, and cell encapsulation.

#### 2.1.2 Chitosan

Chitosan is a naturally occurring polysaccharide obtained by the deacetylation of chitin, which is found in the exoskeletons of crustaceans and insects, as well as fungal cell walls.^[^ ^31^ ^]^ It is a linear polysaccharide composed of *N*‐acetyl glucosamine and *D*‐glucosamine, which exhibit structural similarities to the glycosaminoglycans found in the natural ECM.^[^ ^32^ ^]^ Furthermore, chitosan exhibits remarkable biocompatibility and biodegradability. As a naturally derived polymer, chitosan exhibits inherently low cytotoxicity and is susceptible to enzymatic degradation by lysozyme, among others. Notably, its degradation yields glucosamine, a product that can be safely absorbed or metabolized by the organism. Furthermore, chitosan allows for hydrogel formation through a variety of mild crosslinking methods, including ionic and covalent interactions, as well as gelation triggered by pH or temperature changes. However, chitosan‐based HMs exhibit inherent mechanical brittleness and are prone to structural failure, necessitating combination with complementary materials to improve mechanical stability and durability. After ionic crosslinking, chitosan can coat the surface of alginate HMs to form complete core–shell structure HMs and deliver drugs to BMSCs along the gut–bone axis, reducing the negative effects on senescent cells and restoring the mitochondrial function of BMSCs.^[^ ^33^ ^]^ Furthermore, chitosan HMs integrate with the secondary chemical network, ultimately resulting in the formation of a robust dual‐network 3D‐printed hydrogel.^[^ ^34^ ^]^ Natural chitosan hydrogels exhibit certain limitations, including inadequate solubility. This property necessitates the presence of acidic conditions for dissolution, thereby constraining their compatibility.^[^ ^35^ ^]^ For instance, the injectable chitosan microsphere‐based PLGA–PEG–PLGA hydrogel improves hydrophilicity while concurrently encapsulating exosomes containing vascular endothelial growth factor (VEGF) and dental pulp stem cells, thereby facilitating angiogenesis and bone regeneration.^[^ ^36^ ^]^ More importantly, the chitosan HMs exhibit significant antibacterial activity, which is modulated by a variety of factors. Through the optimization of molecular structure, modification techniques, and fabrication processes, its antibacterial efficacy can be substantially improved, thereby broadening its potential applications across diverse domains.^[^ ^37^, ^38^ ^]^ Consequently, chitosan has the potential to enhance its efficacy through a range of modification and compounding techniques, thereby aligning with diverse application needs.

#### 2.1.3 Collagen

Collagen constitutes the most prevalent protein in the animal kingdom and serves as a fundamental structural element of the ECM, significantly contributing to the preservation of tissue architecture and functionality.^[^ ^39^ ^]^ Collagen HMs exhibit remarkable biocompatibility, degradability, and a composition akin to that of the natural ECM.^[^ ^40^ ^]^ Additionally, they possess favorable mechanical tunability and a high specific surface area, which collectively suggest significant and rapidly advancing potential applications within the biomedical sector.^[^ ^41^ ^]^ 3D‐printed collagen–tannic acid HMs are designed to encapsulate β‐cells, which are capable of secreting insulin in response to extracellular glucose levels, thereby offering a potential therapeutic approach for the management of type 1 diabetes.^[^ ^42^ ^]^ Moreover, a hydrogel matrix composed of hyaluronic acid (HA) and collagen is engineered to encapsulate porous poly(L‐lactide) (PLLA) microspheres. Administered intradermally, this system serves a dual function: filling wrinkles and releasing tranexamic acid to induce a skin‐whitening effect.^[^ ^43^ ^]^ Furthermore, collagen is integral to the regulation of chemotaxis and cellular migration, processes that are essential for effective tissue regeneration.^[^ ^44^ ^]^ Cell adhesion within collagen hydrogels is predominantly facilitated by integrin‐mediated interactions between cells and the ECM. Furthermore, the viscoelastic properties of collagen HMs influence cellular adhesion and function, notably by modulating actin cytoskeletal organization and ROCK‐mediated actin contractility, thereby facilitating cell differentiation.^[^ ^45^ ^]^ Despite its promising advantages, the application of collagen‐based HMs faces several challenges, such as batch‐to‐batch variability in mass production, uncertain long‐term in vivo efficacy, imprecise control over drug release kinetics, and limited targeting efficiency.

#### 2.1.4 Gelatin

Gelatin is a natural product obtained through the partial hydrolysis of animal collagen and is a water‐soluble protein. It possesses advantages such as good biocompatibility, low immunogenicity, and biodegradability. Moreover, it retains multiple bioactive motifs from collagen, including the RGD sequence and matrix metalloproteinase degradable sites.^[^ ^46^ ^]^ These bioactive sequences are essential for supporting cell adhesion, proliferation, and differentiation. Gelatin can be engineered into hydrogels via chemical modification or physical crosslinking, facilitating its broad use in drug delivery and tissue engineering. For example, a microsphere carrier system fabricated from gelatin and a specific binding polysaccharide, when loaded with platelet‐derived growth factor‐BB (PDGF‐BB), can be employed to culture and deliver mesenchymal stem cells (MSCs), thereby enhancing their therapeutic efficacy in tissue regeneration.^[^ ^47^ ^]^ TBA@Gel&Chs microspheres loaded with nucleus pulposus cells (NPCs) effectively promoted the survival and functional recovery of NPCs under oxidative stress by enhancing antioxidant capacity and modulating cellular energy metabolism, providing insights into the treatment of intervertebral disc degeneration (IVDD) using biomaterials combined with cell therapy.^[^ ^48^ ^]^

However, gelatin also has limitations, such as weak mechanical strength and thermoreversibility. To overcome these drawbacks, researchers have modified gelatin through physical or chemical methods. For example, GelMA, obtained via methacrylation modification, not only retains good biocompatibility but also exhibits excellent flexibility and ductility.^[^ ^49^ ^]^ The rapid in vivo degradation of gelatin limits its utility for applications requiring sustained drug release. Conversely, the degradation rate of GelMA can be precisely tuned, rendering it highly suitable for controlled delivery systems. Furthermore, its photocrosslinking capability enables broad applications in 3D bioprinting, tissue engineering, and regenerative medicine. For instance, miR‐17‐5p‐engineered sEVs encapsulated in GelMA hydrogel have been shown to effectively promote the healing of diabetic wounds.^[^ ^50^ ^]^ Utilizing GelMA nanoparticle‐based self‐assembling dual‐crosslinked colloidal gels allows for the integration of 3D printing and hemostasis at bleeding sites, constructing mechanical biomimetic scaffolds for multiple tissues and supporting the functional differentiation of stem cells.^[^ ^51^ ^]^

#### 2.1.5 Hyaluronic Acid

Hyaluronic acid (HA), a native polysaccharide with excellent biocompatibility, biodegradability, and low immunogenicity, forms 3D hydrogel networks upon physical or chemical crosslinking. The resultant hydrogels possess outstanding water absorption and favorable biological properties, which underpin their extensive applications in areas including tissue engineering, controlled drug release, wound healing, joint lubrication, and aesthetic medicine. HA is bioactive and can interact with cells via specific receptors, participating in processes such as cell migration, proliferation, differentiation, inflammatory responses, and wound healing. For instance, CD44‐targeted microbubbles encapsulated within hyaluronic acid hydrogels can localize to the tumor microenvironment (TME) and exhibit reversible responses in contrast‐enhanced pulse sequence signals under ultrasound imaging upon pH changes. This study confirmed the regulatory mechanism of signal variation and crosslinking status on microbubble elasticity, advancing ultrasound medicine from anatomical imaging toward molecular diagnostics.^[^ ^52^ ^]^ Within a 3D hydrogel microenvironment, the efficiency of induced pluripotent stem cell generation is significantly and positively correlated with low‐intensity ultrasound stimulation intensity. The applied ultrasound energy enhances cell membrane fluidity by inducing cytoskeletal reorganization, which in turn promotes HA/CD44 receptor interaction. This activation subsequently initiates the downstream STAT3/AKT signaling cascade, facilitates the mesenchymal‐to‐epithelial transition (MET), and dynamically regulates epigenetic remodeling events, including histone modifications.^[^ ^53^ ^]^

Owing to its inherent bioactivity, exceptional rheological properties, tunable biodegradability, and ease of modification, HA hydrogels find extremely broad applications in the biomedical field. For example, explants based on HA hydrogels can function like “preservation boxes,” effectively maintaining the macroscopic morphology, cell viability, histopathological features, and gene expression of tumor tissues for up to 12 days. They can also accurately predict patient clinical responses to drugs, aiding in the development of more precise personalized treatment plans for patients with peritoneal metastatic cancer.^[^ ^54^ ^]^ An injectable hydrogel based on quaternized carboxymethyl chitosan, oxidized hyaluronic acid, and 3,3′‐dithiobis(propionohydrazide) is formed through dynamic covalent bonds and hydrogen bonding interactions. It exhibits remarkable self‐healing capability, pH responsiveness, good mechanical properties, and excellent cytocompatibility, providing novel ideas and methods for developing next‐generation smart drug delivery systems.^[^ ^55^ ^]^

#### 2.1.6 Cellulose

Cellulose, a natural polysaccharide abundant in plant cell walls, serves as a dietary fiber that promotes intestinal peristalsis, repairs the intestinal barrier, and modulates the gut microbiota. It also exhibits excellent biocompatibility, biodegradability, and renewability. Owing to these unique physicochemical and structural properties, cellulose and its derivatives are widely used in biomedical applications such as drug delivery, tissue engineering, wound healing, and biosensors. Cellulose nanofibers (CNFs), possessing a high specific surface area and good biocompatibility, are also utilized to prepare nanocellulose‐based drug delivery systems. For instance, CNFs exhibit remarkable stiffness and strength, enabling them to withstand the complex physicochemical environment of the gastrointestinal tract. Plant cell‐mimicking carriers assembled from CNFs, pectin, oleic acid, and phospholipids leverage an enzyme‐triggered release mechanism dependent on colonic bacteria to achieve colon‐targeted drug delivery.^[^ ^56^ ^]^

Cellulose hydrogels are hydrophilic materials with a 3D network structure formed through physical or chemical crosslinking using cellulose or its derivatives as the matrix. Owing to their abundant sources, renewability, biodegradability, excellent biocompatibility, and tunable mechanical properties, they demonstrate significant application potential in biomedicine. For example, carboxymethyl cellulose (CMC)‐based pH‐sensitive bionanocomposite hydrogel beads containing 7.5% copper–aluminum layered double hydroxide exhibit optimal sustained drug release performance and can serve as oral antibiotic delivery carriers.^[^ ^57^ ^]^ Porous bionanocomposite hydrogel beads, prepared by coating a zinc‐based metal‐organic framework loaded with the anticancer drug 5‐fluorouracil with pH‐responsive CMC, utilize a synergistic mechanism combining the protective barrier effect in the gastric acid environment and colon‐targeted release. This approach effectively reduces the systemic toxicity of chemotherapeutic drugs and enhances local drug concentration, providing an innovative solution for personalized colorectal cancer treatment and demonstrating significant clinical value.^[^ ^58^ ^]^

#### 2.1.7 Other Natural Materials

Meanwhile, various other natural materials are utilized in the preparation of HMs, including chondroitin sulfate (CS), agarose, fibrin, and silk fibroin. CS is a naturally occurring polysaccharide extensively distributed in tissues including cartilage, skin, and the cornea. It exhibits favorable biocompatibility, biodegradability, and biological functionality.^[^ ^59^ ^]^ The relevant research has developed a biomimetic lubrication microsphere exhibiting immunomodulatory properties. This microsphere was fabricated using microfluidic technology, employing chondroitin sulfate and sericin methacryloyl as the structural framework. Additionally, it was integrated with folic acid‐targeted liposomes, thereby enabling a comprehensive therapeutic approach encompassing lubrication, anti‐inflammatory effects, and tissue repair for the treatment of osteoarthritis.^[^ ^60^ ^]^ Agarose is a linear polysaccharide derived from red algae, consisting of alternating units of D‐galactose and 3,6‐anhydro‐L‐galactose.^[^ ^61^ ^]^ A thermoresponsive agarose HM has been developed for the delivery of tumor cell‐derived microparticles encapsulating methotrexate, aiming to facilitate a synergistic therapeutic approach for malignant ascites through the integration of chemotherapy, photothermal therapy, and immunotherapy.^[^ ^62^ ^]^ Fibrin is a high‐molecular‐weight glycoprotein prized for its excellent biocompatibility and biodegradability, which underpins its extensive use in tissue engineering and regenerative medicine. Nevertheless, despite their considerable porosity and hydrophilicity, fibrin hydrogels suffer from limited mechanical strength. Therefore, they are frequently incorporated with supplementary materials to improve mechanical robustness and overall functionality.^[^ ^63^ ^]^ A fibrin‐based platform for cell microspheres preparation has been developed, which facilitates the self‐assembly of cells into high‐density spheres exceeding the millimeter scale. This system is adaptable to a range of sphere sizes, cell densities, and cell types, demonstrating significant potential for applications in drug screening and tissue engineering.^[^ ^64^ ^]^ Silk fibroin, the core structural protein of silk, consists mainly of repetitive glycine, alanine, and serine sequences. A novel cartilage organoid microsphere fabricated from silk fibroin hydrogel presents an innovative strategy and material platform for cartilage repair. Demonstrating excellent biocompatibility and chondrogenic capacity, this microsphere effectively promotes in vivo cartilage regeneration, thereby offering a promising therapeutic avenue for degenerative cartilage conditions such as osteoarthritis.^[^ ^65^ ^]^

In summary, the natural materials used in HMs exhibit favorable biocompatibility and biodegradability, rendering them suitable for diverse biomedical applications. Nonetheless, these materials typically possess limited mechanical strength and stability, often necessitating chemical modification or integration with other substances to improve their functional performance.

### 2.2 Synthetic Material

Synthetic materials represent a significant category of HMs. These materials are produced via chemical synthesis techniques and exhibit features such as tunable structural properties, adjustable performance parameters, high mechanical strength, and enhanced stability. These attributes address certain limitations inherent in natural materials. In this section, we explore various types of synthetic materials that have been used in the preparation of HMs, including poly(lactic*‐*co‐glycolic acid) (PLGA), poly(ethylene glycol) diacrylate (PEGDA), polyvinyl alcohol (PVA), polycaprolactone (PCL), poly(*N*‐isopropylacrylamide) (PNIPAM), and other synthetic materials (polyethylene glycol (PEG) and polyacrylamide (PAM)).

#### 2.2.1 PLGA

PLGA, the most widely used synthetic biodegradable polymer in biomedicine, is formed by random copolymerization of two monomers: lactic acid (LA) and glycolic acid (GA). This material degrades into LA and GA monomers, ultimately metabolizing into carbon dioxide and water without toxic side effects. Owing to its tunable degradation rate, excellent biocompatibility, and safety profile, PLGA serves as a core material for drug delivery, tissue engineering, and advanced medical devices. For instance, in a mouse model of retinal ischemia–reperfusion injury, a PLGA microcapsule system loaded with MSC‐derived exosomes, when injected intravitreally, localized beneath the vitreous cavity and sustained exosome release, restoring the thickness of the retinal outer nuclear layer to normal levels.^[^ ^66^ ^]^ A cryogenically 3D‐printed magnesium peroxide (MgO_2_)–PLGA nanocomposite scaffold enables precise spatiotemporal control over the release of ROS and magnesium ions. This system remodels the antitumor immune microenvironment by activating macrophage M1 polarization and concurrently promoting the osteogenic differentiation of BMSCs, achieving synergistic tumor suppression and bone regeneration for improved postsurgical management of bone tumors.^[^ ^67^ ^]^

#### 2.2.2 PEGDA

PEGDA is a versatile polymeric material with broad application prospects, synthesized as a bifunctional monomer through chemical reactions between poly(ethylene glycol) (PEG) and acrylate groups. The acrylate groups at both ends of its molecular structure can undergo free radical polymerization to form 3D‐crosslinked networks. PEGDA exhibits excellent biocompatibility, water solubility, and photoresponsive properties, with its biocompatibility primarily attributed to the PEG segments, making it an ideal material for bioengineering applications. Furthermore, its acrylate groups allow crosslinking under specific conditions to form stable hydrogel networks. For example, a highly adhesive, biodegradable, soft, and stretchable PEGDA–gelatin hydrogel demonstrates superior compatibility with dynamic moist cavities such as the nasal cavity, offering a novel strategy for sustained drug delivery in allergic rhinitis treatment and hemostasis of epistaxis.^[^ ^68^ ^]^ Silicate nanoplatelet (SN)‐reinforced GelMA–PEGDA hydrogels release degradation products that provide essential trace elements for angiogenesis and osteogenesis, effectively promoting vascular and bone tissue regeneration, thus offering valuable insights for designing bone regeneration materials.^[^ ^69^ ^]^

#### 2.2.3 PVA

PVA is a water‐soluble polymer characterized by its outstanding biocompatibility, nontoxicity, lack of immunogenic response, and its chemical stability.^[^ ^70^ ^]^ PVA hydrogels are extensively utilized in drug delivery systems and tissue engineering applications owing to their superior biocompatibility, porous architecture, and tunable mechanical characteristics.^[^ ^71^ ^]^ PVA HMs are extensively utilized as embolic agents in clinical settings owing to their low density, high elasticity, and favorable biocompatibility, thereby establishing them as a significant option for transarterial radioembolization therapy. The imaging properties of PVA microspheres, combined with their capacity for chemotherapeutic drug loading and the advancement of radiolabeling techniques, offer substantial benefits for the personalized management of patient therapy.^[^ ^72^ ^]^ Furthermore, microglia‐targeting RAP12 peptide‐modified interleukin‐4 nanoparticles (RIL4) were encapsulated into borate‐functionalized PVA HMs via a biotin–avidin binding strategy. In a murine model of ischemic stroke, this microsphere system significantly reduced cerebral atrophy, restored neurobehavioral function, and promoted vascular endothelial cell proliferation alongside neuronal differentiation of neural stem cells. These results confirm its dual mechanism in promoting neural repair by modulating the immune‐neurovascular network.^[^ ^73^ ^]^ Nevertheless, the inadequate thermal stability and restricted functional properties of PVA hinder its broader utilization. In order to address these issues, the researchers use composite materials.^[^ ^74^ ^]^ For example, the hydrogel facilitates glucose‐responsive insulin release and exhibits anti‐inflammatory effects via borate ester bond crosslinking between polylysine–boric acid (PL–PBA) and PVA.^[^ ^75^ ^]^ In addition, oxygen nanobubbles were encapsulated within exosomes and subsequently integrated into a composite hydrogel composed of polyvinyl alcohol and gelatin. The intrinsic self‐healing characteristics of the hydrogel, along with the hemostatic properties of gelatin, contribute to effective hemorrhage control. More importantly, the crosslinked network within the hydrogel facilitates the catalytic decomposition of hydrogen peroxide, thereby enhancing the mitigation of wound inflammation.^[^ ^76^ ^]^ Therefore, PVA HMs represent a class of platform materials characterized by well‐balanced properties and superior biocompatibility. Their primary advantage is attributed to their safety profile; however, significant challenges remain regarding their degradability and drug loading efficiency for specific pharmaceutical agents.

#### 2.2.4 PCL

PCL is a semicrystalline, biodegradable thermoplastic polyester distinguished by its excellent biocompatibility and degradability. Additionally, it effectively facilitates collagen synthesis within tissues, rendering it a highly suitable material for applications in tissue filling.^[^ ^77^, ^78^ ^]^ The capacity of PCL microspheres to promote the formation of collagen septa within adipose tissue holds considerable importance for their utilization in both aesthetic and reconstructive medical applications.^[^ ^79^ ^]^ Furthermore, the 3D‐printed PCL scaffolds integrated with injectable hydrogels composed of sodium alginate and magnesium‐doped mesoporous bioactive glass microspheres have been employed for meniscal tissue regeneration.^[^ ^80^ ^]^ However, PCL has strong hydrophobicity, which leads to low encapsulation efficiency for hydrophilic drugs. Furthermore, the hydrophobic characteristics of the microsphere surface may influence its interactions with cells, while the extended degradation period presents a potential risk of prolonged residual presence. Consequently, the functionality is enhanced via modification by incorporating elastic porous poly(*l*‐lactide*‐co*‐ε‐caprolactone) (PLCL) microspheres into ECM hydrogels, forming injectable composites that enable the dual release of interleukin‐4 (IL‐4) and insulin‐like growth factor‐1 (IGF‐1).^[^ ^81^ ^]^ PCL microspheres represent a notable platform in biomedical materials, emerging as a distinct candidate for sustained drug delivery and injectable tissue engineering scaffolds. Future research may focus on surface modification strategies and the development of intelligent PCL composite microspheres responsive to specific stimuli such as pH, temperature, or enzymatic activity.

#### 2.2.5 PNIPAM

PNIPAM HMs are widely used in biomedical applications due to their distinctive temperature‐responsive properties. Specifically, these microspheres exhibit water absorption and swelling behavior at temperatures below a critical threshold (32 °C), whereas they undergo dehydration and contraction when exposed to temperatures above 32 °C.^[^ ^82^, ^83^ ^]^ For example, it can function as a drug delivery system by regulating drug release in response to temperature variations, thereby enabling controlled drug release and targeted delivery.^[^ ^84^ ^]^ Relevant research has developed a wearable intelligent wound dressing integrating wound exudate management, sensor‐based monitoring, closed‐loop therapeutic delivery, and flexible circuitry components. In the context of therapeutic delivery, the heat generation function of thermosensitive PNIPAM HMs within dressings is regulated through the activation and deactivation of liquid metal coils.^[^ ^85^ ^]^ Nevertheless, PNIPAM exhibits limited biodegradability, suboptimal mechanical strength, a singular responsive behavior, and restricted functional capabilities. Pressure‐sensitive adhesive functions as a reticular scaffold integrated within the PNIPAM network, endowing the hydrogels with a stable volume, tunable mechanical properties in situ, and superior adhesion characteristics responsive to varying temperature conditions.^[^ ^86^ ^]^ Furthermore, the incorporation of acrylamide into the PNIPAM hydrogel to synthesize poly(*n*‐isopropylacrylamide*‐co*‐acrylamide) enables the modulation of the lower critical solution temperature. This adjustment facilitates the customization of thermal response to suit various application contexts that necessitate functionality across distinct temperature intervals.^[^ ^87^ ^]^ PNIPAM exhibits significant potential for applications within the biomedical domain; however, its utility is constrained by inherent limitations, including insufficient mechanical strength and limited biodegradability. Consequently, contemporary research efforts are primarily directed toward enhancing its properties through composite formation with other materials, such as the incorporation of inorganic nanoparticles or functionalized polymers.

#### 2.2.6 Other Synthetic Materials

Synthetic materials are widely used in preparing HMs due to their tunable properties, such as molecular weight, crosslinking density, and mechanical strength, such as polyethylene glycol (PEG) and polyacrylamide (PAM). PEG, a flexible and hydrophilic linear polymer, forms a 3D network structure through crosslinking, which exhibits high water absorption capacity, biocompatibility, and tunable mechanical properties. PEG HMs can have their functionalities augmented via surface modification or functionalization techniques, including the incorporation of copolymers, peptides, or fluorescent labels. Such modifications can enhance properties such as lubricity, antibacterial activity, and targeted delivery potential.^[^ ^88^ ^]^ PAM serves as an optimal substrate for the fabrication of HMs. By employing molecular design strategies and technological advancements, it is possible to synthesize a range of PAM‐based HMs exhibiting tunable properties and multifunctional capabilities. The prodrug system capitalizes on the synergistic interaction between the zeolite imidazolate framework‐8 and polyacrylamide HMs, which are loaded with indole‐3‐acetic acid. This combination markedly enhances ROS generation and subsequent antibacterial efficacy, thereby facilitating the accelerated healing of infected wounds.^[^ ^89^ ^]^ Notably, various synthetic materials have different properties and biomedical applications.

Conclusively, in the fabrication of HMs, synthetic materials often exhibit lower biocompatibility and biodegradability compared to natural materials, thereby restricting their applicability in specific domains. Additionally, the synthesis and modification procedures associated with synthetic materials tend to be more intricate and expensive. Furthermore, the integration of synthetic and natural materials, such as sodium alginate and gelatin, has been extensively investigated to achieve an optimal balance between functional performance and biocompatibility.

## 3 Preparation Methods

Various methods are available for preparing HMs (**Table** **1**), such as emulsification, microfluidics, electrohydrodynamic spraying, lithography, 3D bioprinting, and other techniques (e.g., mechanical fragmentation and aerodynamic microfluidics). Each method offers distinct advantages and faces specific limitations. The selection of an appropriate technique should be dictated by the intended application, the required microsphere characteristics, and production cost considerations.

**Table 1:** Comparison of HM preparation methods.

Preparation methods	Particle size uniformity	Yield	Cost	Advantages	Limitations
Emulsification	Low	High	Low	Easy operation, efficient, easy regulation of process parameters	Solvent residues, uneven dispersion of emulsion, wide particle size distribution
Microfluidic	High	High	High	Uniform morphology and size, highly tunable parameters, excellent reproducibility	High equipment maintenance costs, operational complexity, prolonged processing times
Electrohydrodynamic spraying	High	Low	High	Uniform size, high encapsulation, efficiency, minimal damage to cells	Low yield, operational complexity, poor monodispersity
Lithography	High	Low	High	Precise control, specific shapes, wide material adaptability	Narrow applicability, elevated costs, poor efficiency
3D bioprinting	High	Low	High	High precision, extensive customizability, broad applicability, excellent controllability	Complex equipment parameters, prolonged production times, elevated costs
Mechanical fragmentation	Low	High	Low	Unparalleled simplicity, low cost, high yield	Poor biocompatibility, low size uniformity
Aerodynamic microfluidics	High	High	High	Efficient, clean, high biocompatibility	Difficult to generate complex structures

### 3.1 Emulsification

Emulsification is a process that disperses one immiscible liquid into another to form kinetically stable droplets, facilitated by surfactants or solid particles that adsorb at the interface to reduce interfacial tension and form protective films.^[^ ^90^ ^]^ These droplets serve as templates for HMs upon crosslinking.

Depending on structural complexity, emulsification techniques can be divided into two main types: single emulsions (W/O and O/W) and double emulsions (W/O/W). The W/O system disperses an aqueous polymer solution such as alginate and chitosan into a continuous oil phase such as mineral oil containing Span 80.^[^ ^91^, ^92^, ^93^ ^]^ This interfacial film, formed by the alignment of surfactant molecules, prevents droplet coalescence. Due to the cytocompatibility of the oil phase, this system is particularly suitable for hydrophilic drug encapsulation and cell microencapsulation. For example, microcapsules of W/O‐emulsified alginate have exhibited excellent biocompatibility in bone regeneration, maintaining over 90% viability of encapsulated human amnion.^[^ ^94^ ^]^ In contrast, the O/W system disperses hydrophobic drugs such as paclitaxel, which are dissolved in an oily phase such as PLGA, into an aqueous continuous phase.^[^ ^95^ ^]^ Microspheres with controlled release kinetics are typically obtained through solidification induced by solvent evaporation. This system enables controlled release of lipophilic drugs, as demonstrated by a microfluidic‐based O/W system producing paclitaxel‐loaded PLGA nanoparticles camouflaged with cancer cell membranes, which achieved 73% tumor growth inhibition in lung cancer models.^[^ ^96^ ^]^ The W/O/W architecture is created by first dispersing an aqueous biomolecule phase into an oil phase, forming a primary W/O emulsion. This primary emulsion is then redispersed into an external aqueous phase, forming a concentric “water–oil–water” structure. This system efficiently encapsulates sensitive biomolecules such as proteins due to the layered interfacial barriers, preventing denaturation and leakage, while supporting controlled release kinetics for sustained or targeted drug delivery.^[^ ^97^, ^98^, ^99^ ^]^ While the emulsification technique is relatively simple to implement and allows for easy adjustment of process parameters, it presents a significant challenge in controlling microsphere size uniformity. This drawback subsequently contributes to problems including solvent retention, low recovery yield, and higher purification expenses.

### 3.2 Microfluidic

Microfluidic technology provides precise control over hydrogel microsphere fabrication, enabling superior regulation of size, morphology, and functionality compared to conventional emulsification methods.^[^ ^100^ ^]^ This technique utilizes engineered microchannels such as T‐junctions and flow‐focusing geometries to produce monodisperse droplets through shear forces, followed by crosslinking using ultraviolet irradiation, chemical agents, or thermal stimuli.^[^ ^101^, ^102^ ^]^ Fluorinated oils such as SF33 combined with photoinitiators accelerate droplet solidification, maintaining uniformity and preserving cell viability during encapsulation.^[^ ^103^ ^]^

The principal advantages of this method lie in its ability to fine‐tune droplet size through controlled adjustments of flow rates and surfactant concentrations, coupled with the seamless integration of platforms that support continuous processes from droplet formation and gelation to final purification. For example, T‐junction devices with blue‐light curing enable precise volume control and uniform gelation, while volatile fluorinated oils permit direct microsphere transfer to culture media, eliminating washing steps.^[^ ^104^, ^105^ ^]^ In T‐junction systems, increasing continuous phase flow rates decreases droplet size, whereas elevated surfactant concentrations enhance stability and uniformity.^[^ ^106^ ^]^ Such control is essential for drug delivery, ensuring consistent release kinetics, and tissue engineering, regulating cellular responses through mechanical properties.^[^ ^107^ ^]^

Microfluidic systems maintain cell viability and functionality under mild conditions, as shown by alginate–gelatin copolymers sustaining HepG2 cell activity long‐term.^[^ ^108^ ^]^ Moreover, microfluidics facilitates the production of functionalized HMs for applications in targeted therapies. The matrix can incorporate bioactive molecules such as growth factors and anticancer drugs to enable targeted therapies and facilitate therapeutic efficacy.^[^ ^109^ ^]^ Specifically, drug‐loaded microspheres enable localized cancer therapy, reducing systemic toxicity and improving therapeutic efficacy.^[^ ^110^ ^]^ Ligand‐functionalized microspheres also permit targeted cell capture and isolation for diagnostics.^[^ ^111^ ^]^ Consequently, microfluidic technology enables the fabrication of microspheres exhibiting uniform morphology and size, characterized by highly tunable parameters and excellent reproducibility. Nonetheless, this technique is associated with elevated production costs, operational complexity, and prolonged processing times.

### 3.3 Electrohydrodynamic Spraying

Electrospray technology leverages electrofluidodynamics to produce monodisperse HMs by atomizing polymer solutions under an electric field, enabling precise control over size, morphology, and functionality.^[^ ^112^ ^]^ The process involves high‐voltage‐induced Taylor cone formation, where Coulombic forces overcome surface tension to generate uniform droplets.^[^ ^113^ ^]^ The technique's adaptability to polymers like chitosan, PEGDA, and PVA allows fabrication of biocompatible, pH‐sensitive microgels with tunable properties via voltage, concentration, and flow rate adjustments.^[^ ^114^, ^115^ ^]^ For instance, chitosan‐based microspheres exhibit lysozyme‐degradable structures suitable for drug delivery and cell encapsulation.^[^ ^116^ ^]^

One of the primary benefits of this method is its capacity to encapsulate sensitive biological entities, ensuring their viability is not compromised. Specifically, the solvent‐free process preserves cell integrity and protein activity, enabling long‐term culture of stem or tumor cells.^[^ ^117^ ^]^ Beyond maintaining biological activity, integration with photocrosslinking further accelerates droplet solidification, enhancing applications in tissue engineering.^[^ ^118^ ^]^ Moreover, multifunctional systems, such as heparin‐functionalized PVA microspheres, combine therapeutic delivery with regenerative capabilities.^[^ ^119^ ^]^ Coaxial electrospraying expands this potential by creating hollow or core–shell structures for hydrophobic drug encapsulation.^[^ ^120^ ^]^ Electrohydrodynamic spraying enables the production of microspheres exhibiting high size uniformity and a narrow distribution. Nevertheless, this technique suffers from low yield and operational complexity. It is also unsuitable for processing high‐viscosity solutions, primarily because the jet formation requires overcoming significant electrostatic stresses.

### 3.4 Lithography

Lithography is a light‐directed patterning technique that enables the fabrication of HMs with sub‐100 nm resolution and complex geometries, such as anisotropic shapes and porous networks.^[^ ^121^ ^]^ By integrating photochemical crosslinking with physical or digital masks, lithography allows precise tailoring of hydrogel microsphere size, shape, and surface chemistry, making it particularly suitable for biomedical applications requiring reproducibility and uniformity.^[^ ^122^, ^123^ ^]^ The integration of lithography with microfluidics further expands its utility by enabling continuous HMs synthesis with controlled anisotropy and size distribution, ideal for high‐throughput production.^[^ ^124^, ^125^ ^]^ Notably, stop‐flow lithography improves upon traditional photolithography by combining intermittent UV exposure with continuous flow.^[^ ^126^ ^]^ This method ensures geometric precision during large‐scale production, a crucial feature for generating uniform cell spheroids that faithfully mimic native tissue architecture in regenerative medicine.

Beyond structural control, lithography excels in engineering stimuli‐responsive HMs that respond to environmental cues such as pH, temperature, and enzymatic activity through the incorporation of functional nanomaterials into the hydrogel matrix. These systems enable sustained release of therapeutic agents, improving treatment efficacy for diseases like cancer and bacterial infections, while precise spatial control over nanoparticle distribution supports multifunctional designs capable of simultaneous diagnostics and therapy.^[^ ^127^ ^]^ Furthermore, lithography is compatible with natural polymers such as gelatin and hyaluronic acid, which offer excellent biocompatibility and biodegradability.^[^ ^128^ ^]^ These materials can be fabricated into microspheres with tunable mechanical properties for wound healing and tissue regeneration, and their surfaces can be functionalized with bioactive molecules such as peptides and growth factors to enhance cell adhesion, proliferation, and differentiation.^[^ ^10^, ^129^ ^]^ Lithography enables excellent precision and resolution in HM fabrication, rendering it highly suitable for applications demanding exact control over microsphere architecture. Nevertheless, its scalability remains limited by substantial operational costs, low production throughput, and a dependence on specialized instrumentation.

### 3.5 3D Bioprinting

3D bioprinting is a computer‐aided, layer‐by‐layer technique that deposits bioinks to create 3D structures with tunable mechanical and biological properties. HMs fabricated from biocompatible polymers such as GelMA and HA collectively resolve critical challenges in tissue engineering by preserving cell viability, ensuring mechanical stability, and recapitulating ECM‐like microenvironments.^[^ ^130^, ^131^ ^]^ Precisely tailoring hydrogel microsphere size, composition, and porosity further optimizes nutrient diffusion and cell infiltration.^[^ ^132^, ^133^ ^]^

A major innovation in this field is the use of jammed microgel inks, which exhibit shear‐thinning behavior to enable smooth extrusion and rapid structural recovery after printing.^[^ ^134^ ^]^ These inks can be reinforced through secondary crosslinking to enhance mechanical integrity, facilitating the fabrication of multimaterial constructs with anatomical precision. For instance, GelMA‐based microspheres have been printed into lumen‐like structures with osteogenic differentiation potential, while HA microgels functionalized with thiol groups and crosslinked using PEG acrylate demonstrate tunable stiffness and improved cell adhesion.^[^ ^135^, ^136^, ^137^ ^]^ Additionally, submerged bioprinting, a technique utilizing high‐density fluorocarbon liquids, has emerged to support the fabrication of high‐aspect‐ratio constructs such as vascular grafts and branching networks.^[^ ^138^, ^139^ ^]^ This method overcomes the mechanical instability commonly associated with traditional hydrogel printing by leveraging buoyancy to maintain shape fidelity during deposition.^[^ ^140^, ^141^ ^]^ The integration of bioactive components, such as growth factors and adhesion peptides, further enhances cellular responses, promoting proliferation and differentiation for regenerative medicine applications.^[^ ^142^, ^143^ ^]^ The fabrication technique of 3D‐printed HMs offers several benefits, including high precision, extensive customizability, and broad applicability. However, it is also associated with certain limitations, such as prolonged production times, the requirement for sophisticated equipment, and elevated costs. In comparison to alternative fabrication methods, 3D printing demonstrates distinct advantages in producing complex structures and enabling multimaterial integration. Nonetheless, further improvements are necessary to enhance its resolution and operational efficiency.

### 3.6 Other Methods

Furthermore, several alternative techniques exist for the preparation of microspheres, such as mechanical fragmentation and aerodynamic microfluidics. Mechanical fragmentation entails the physical shearing or grinding of bulk hydrogels to produce particles on the micron scale. This technique represents a relatively straightforward and direct approach, appropriate for the rapid fabrication of microspheres. Nonetheless, akin to emulsification and electrohydrodynamic spraying, this method exhibits a broad size distribution and limited precision in achieving uniformity, thereby diminishing its efficacy in encapsulating cells or drugs.^[^ ^144^ ^]^ In aerodynamic microfluidics, HM fabrication fundamentally relies on airflow to generate shear forces and dynamic effects that atomize the liquid stream. This approach allows precise regulation of droplet formation and dispersion, enabling the production of highly uniform HMs.^[^ ^145^ ^]^ Previous studies have documented the fabrication of microspheres composed of GelMA, alginate methacrylate (AlgMA), and osteogenic membrane vesicles utilizing aerodynamic microfluidics combined with photopolymerization, followed by in situ premineralization to enhance the reconstruction of bone cell networks and promote targeted bone regeneration.^[^ ^146^ ^]^

In summary, there are various methods for preparing HMs, and each method has its unique advantages and disadvantages. The preparation method of HMs constitutes a fundamental aspect of their application. Various preparation approaches critically influence essential characteristics, including particle size, monodispersity, encapsulation efficiency, biocompatibility, and overall production cost. Consequently, the most appropriate method can be selected based on specific individual requirements.

## 4 Biomedical Applications

### 4.1 Drug Delivery

**Figure 2:**
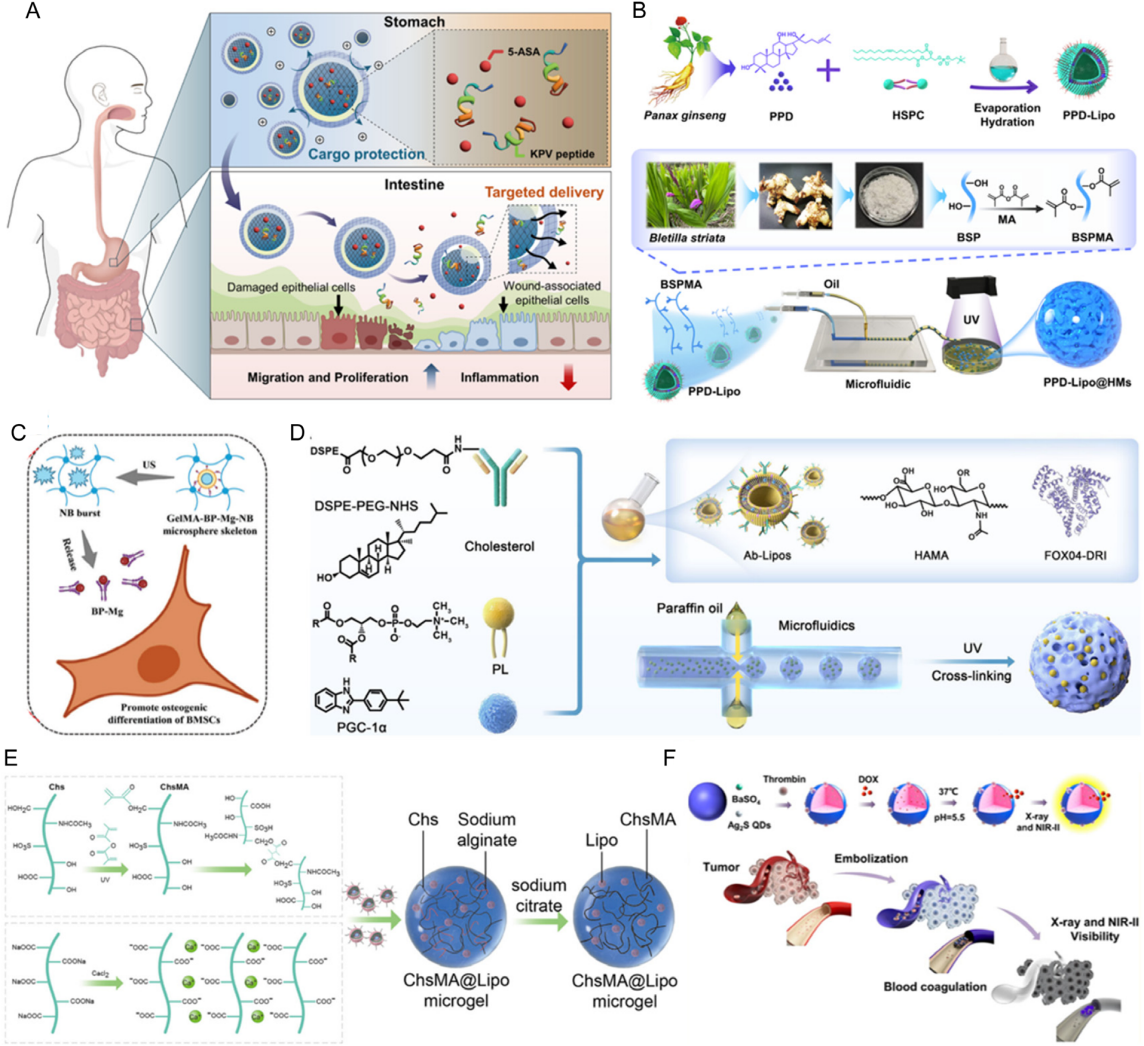
HMs for drug delivery. A) HMs as carriers for drug delivery of small‐molecule pharmaceuticals. The multicompartmental HMs maintain the anti‐inflammatory efficacy of 5‐ASA and KPV in stomach conditions while sustaining their therapeutic effects on gut epithelial cell migration and proliferation. Reproduced with permission.^[^ ^148^ ^]^ Copyright 2025, American Chemical Society. B) HMs as carriers for drug delivery of Chinese medicine. The synthesis process and preparation of PPD‐Lipo@HMs. Reproduced with permission.^[^ ^149^ ^]^ Copyright 2025, Springer Nature. C) HMs as carriers for drug delivery of metal ions. HMs were triggered ion release process. Reproduced with permission.^[^ ^150^ ^]^ Copyright 2025, John Wiley & Sons. D) HMs as carriers for drug delivery of peptides. Preparation of HAMA/FOXO4‐DRI/Ab‐Lipos HMs. Reproduced with permission.^[^ ^151^ ^]^ Copyright 2025, John Wiley & Sons. E) HMs as carriers for drug delivery of liposomes. Schematic illustration of the synthetic process of ChsMA@Lipo via double hinges. Reproduced with permission.^[^ ^59^ ^]^ Copyright 2022, Elsevier. F) HMs as carriers for drug delivery of quantum dots. A gelatin microsphere embolization material that simultaneously encapsulates Ag_2_S QDs and BaSO_4_. Reproduced with permission.^[^ ^152^ ^]^ Copyright 2023, American Chemical Society.

Drug delivery systems can facilitate drug loading and release, enhancing the concentration and utilization of therapeutic agents at targeted lesion sites, and have emerged as a prominent area of research within the domain of biological tissue engineering in recent years. HMs have gained recognition as a novel and effective drug delivery system, attributed to their superior biocompatibility and customizable characteristics.^[^ ^147^ ^]^ HMs of suitable dimensions can offer significant protection for drugs, thereby reducing the rapid clearance of these agents by vascular or lymphatic systems. Recent studies have increasingly focused on the application of HMs as carriers for drug delivery.^[^ ^90^ ^]^ HMs possess the capability to encapsulate a variety of substances, including small‐molecule pharmaceuticals, Chinese medicine, metal ions, peptides, liposomes, and quantum dots (**Table** **2**). These microspheres serve diverse functions in the treatment of various diseases. For instance, the multicompartmental HMs retain the anti‐inflammatory activity of 5‐aminosalicylic acid and Lys‐Pro‐Val under gastric acid conditions, effectively promoting the migration and proliferation of colonic cells and enhancing the overall efficacy of drug treatment in inflammatory bowel disease (**Figure** **2**A).^[^ ^148^ ^]^ In addition, the microfluidic‐engineered HMs that are encapsulated with Bletilla striata polysaccharides and protopanaxadiol have been shown to enhance angiogenesis, modulate the immune microenvironment in diabetic wounds, diminish inflammatory responses, and consequently expedite the healing process (Figure 2B).^[^ ^149^ ^]^ Very recently, the ultrasound‐triggered HMs loaded with Mg^2+^ and bisphosphonates have achieved precise controlled release of Mg^2+^, ultimately effectively promoting bone reconstruction (Figure 2C).^[^ ^150^ ^]^ Furthermore, the FOXO4‐DRI peptide was integrated with the HAMA hydrogel through microfluidic technology and subsequently conjugated to immune liposomes with surface‐modified AQP1 antibody and encapsulated PGC‐1α agonists ZLN005, thereby facilitating the precise modulation of the life cycle of senescent cells for enhancing mitochondrial biogenesis (Figure 2D).^[^ ^151^ ^]^ Moreover, chondroitin sulfate‐based microspheres conjugated with drug‐loaded liposomes demonstrate a dual antioxidant mechanism for osteoarthritis therapy. First, the combined lipid films and hydrogel matrix enable sustained release of the encapsulated antioxidant drugs. Second, the microspheres themselves can be enzymatically degraded into chondroitin sulfate monomers, which directly scavenge ROS (Figure 2E).^[^ ^59^ ^]^ Another study demonstrated that gelatin microspheres effectively encapsulate silver sulfide quantum dots (Ag2S QDs), barium sulfate (BaSO_4_), doxorubicin, and thrombin, serve as a viable medium for drug delivery, and exhibit significant potential for applications in arterial embolization and hemostasis (Figure 2F).^[^ ^152^ ^]^

**Table 2:** Comparison of HMs for drug delivery.

Drug	Materials	Methods	Disease	Mechanism	Reference
Small‐molecule pharmaceuticals	PEGDA	Microfluidic	Inflammatory bowel disease	Preserve the anti‐inflammatory activities of 5‐aminosalicylic acid and Lys‐Pro‐Val, promote the migration and proliferation of colonic cells	[148]
Chinese medicine	Bletilla striata polysaccharide	Microfluidic	Diabetic wound	Regulate the immune microenvironment, promote angiogenesis, promote cell migration and proliferation, increase collagen deposition	[149]
Metal ions	GelMA	Microfluidic	Osteoporosis	Penetrate the cancellous bone region, trigger the targeted controlled release of magnesium ions through ultrasound	[150]
Peptides	HAMA	Microfluidic	IVDD	Restore the p53/BCL‐2/caspase‐3 signaling pathway, promote the removal of late senescent cells, reduce the production of senescence‐associated secretory phenotype factors	[151]
Liposomes	Chondroitin sulfate	Electrohydrodynamic spraying	Osteoarthritis	Exert a dual antioxidant effect, maintain a metabolic balance of the chondrocytes, inhibit the activation of downstream NF‐κB and MAPK pathways	[59]
Quantum dots	Gelatin	Emulsification	Embolization	Double luminescence, immobilize thrombin on the surface, load antitumor drugs inside, potential embolization system for drug delivery	[152]

More importantly, HMs exhibit capabilities for targeted delivery, responsiveness to environmental stimuli, and multifunctional applications. For example, HMs rapidly target M1 macrophages with the help of folic acid and quickly enter the interior of the cells through folic acid receptor‐mediated endocytosis, achieving in situ accumulation of drugs at the target site. Therefore, using M1 macrophages as drug targets is of great significance for the treatment of osteoarthritis.^[^ ^153^ ^]^ In addition, the pertinent research has effectively developed a bilayer HMs that incorporate three functionalities for oral administration: pH responsiveness, time delay, and degradation by colonic enzymes. Combining the dual biological effects of curcumin in reducing inflammation and promoting the repair of colonic mucosal damage, it is targeted and released in the colon based on the colonic microenvironment.^[^ ^154^ ^]^ The pH‐responsive microspheres represent “smart” drug delivery systems that enable on‐demand therapeutic release, thereby enhancing treatment specificity and efficacy while minimizing off‐target effects. Their design relies on incorporating responsive functional groups, yet a key challenge for antitumor applications lies in ensuring activation exclusively within the acidic TME, avoiding unintended triggering in other slightly acidic physiological regions.^[^ ^20^ ^]^ Particularly noteworthy are the double‐positively charged functional HMs, which possess the capabilities of targeting the ECM of chondrocytes, facilitating cartilage penetration, and promoting cellular phagocytosis. These microspheres hold significant promise for therapeutic applications in the treatment of osteoarthritis and exhibit considerable potential for use in the regeneration of various other tissues.^[^ ^155^ ^]^

Despite the significant promise of HMs in drug delivery, their clinical translation still faces several challenges. In particular, the fabrication of HMs requires precise control over crosslinking density and drug loading capacity to ensure both biocompatibility and desirable drug release profiles. Further research is also needed to address concerns related to long‐term stability and controlled biodegradability of these microspheres.

### 4.2 Cell Culture

HMs, utilized as carriers for cell culture, signify a transformative advancement in the domains of biomedical engineering, tissue engineering, and regenerative medicine.^[^ ^144^ ^]^ These microspheres address numerous constraints associated with conventional 2D culture systems by offering a scaffold that more accurately mimics the 3D microenvironment found in vivo. Consequently, they enhance cellular behavior, functionality, and the overall validity of research outcomes. Furthermore, HMs offer several advantages, including the provision of a 3D culture environment that closely resembles the natural ECM found in vivo.^[^ ^156^ ^]^ These microspheres function by mimicking a 3D ECM, facilitating critical cellular processes such as adhesion, migration, proliferation, and differentiation, thereby enabling complex cell–matrix interactions. A key advantage is their spherical morphology, which provides a large specific surface area for high‐density cell loading within a small volume, dramatically increasing both culture efficiency and final cell yield.^[^ ^157^ ^]^ The porous architecture facilitates the efficient diffusion of nutrients and oxygen into the interior of the microspheres while simultaneously enabling the effective removal of metabolic waste products, thereby maintaining the viability of the internal cellular environment.^[^ ^158^ ^]^ Meanwhile, the hydrogel network can conveniently introduce bioactive molecules to achieve precise regulation of cell behavior. Therefore, the delivery of cells via HMs has emerged as a hotspot of research in the therapeutic method of a range of diseases.

**Figure 3:**
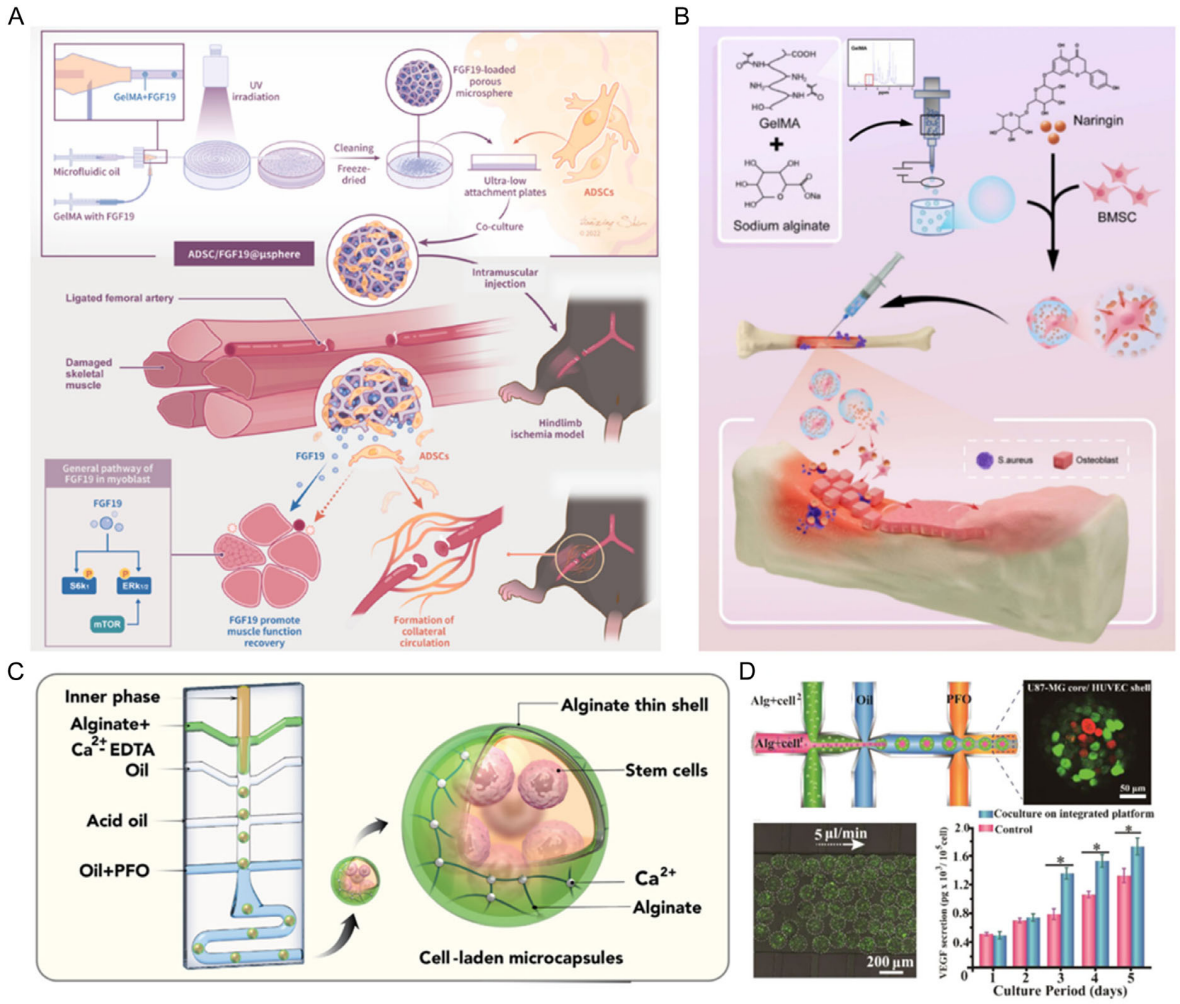
HMs for cell culture. A) Schematic illustration of the synthetic process of ADSC/FGF19@μspheres and ischemic limb restoration. Reproduced with permission.^[^ ^161^ ^]^ Copyright 2023, KeAi. B) HMs loaded with cells and drugs control infection and promote healing of osteomyelitis. Reproduced with permission.^[^ ^162^ ^]^ Copyright 2023, IOP Publishing. C) The preparation process of core–shell alginate microcapsules to encapsulate MSCs. Reproduced with permission.^[^ ^164^ ^]^ Copyright 2024, Cell Press. D) Schematic illustration of the modified first PDMS module and fabrication of the specially assembled heterogeneous‐cell‐laden microspheres. Reproduced with permission.^[^ ^165^ ^]^ Copyright 2019, American Chemical Society.

The methods for loading cells onto HMs can be primarily classified into two categories: the surface strategy and the internal strategy.^[^ ^159^ ^]^ In the surface strategy, HMs featuring an innovative crater‐like topography have the capacity to promote nonspecific protein adsorption on their surfaces, thereby enhancing their affinity for cells. HMs surface‐coated with HUVECs via self‐assembly collagen fibers by synthesized utilizing bioprinting technology were subcutaneously injected into immunodeficient mice and demonstrated rapid angiogenesis and functional anastomosis with host vessels.^[^ ^160^ ^]^ Using microfluidic technology, we developed GelMA microspheres loaded with the myogenic cytokine FGF19 and subsequently adhered adipose‐derived stem cells (ADSCs) into their porous network, thereby establishing a codelivery system for combined cell and cytokine therapy. This system ensures precise ADSC implantation and retention at the lesion site, coupled with sustained local FGF19 release, which collectively promotes myoblast recruitment, differentiation, and myofibril growth in the ischemic region (**Figure** **3**A).^[^ ^161^ ^]^ Furthermore, relevant researchers successfully prepared naringin GelMA/alginate microspheres of different particle sizes by using electrospray microprocessing technology. The results showed that the microspheres with relatively smaller diameters (200 μm) were more conducive to the initial adhesion, growth, spreading, and osteogenic differentiation of BMSCs and could effectively treat tibial osteomyelitis in rats, with clinical application prospects (Figure 3B).^[^ ^162^ ^]^ In addition, the injectable system based on HMs was employed for the cultivation of chondrocytes, thereby illustrating its potential applicability in the field of cartilage tissue engineering.^[^ ^163^ ^]^

In the internal strategy, relevant researchers have prepared stiff‐shell alginate microsphere using microfluidic technology and controlled the mechanical properties of the shell by adjusting the molecular weight of alginate. The author found that stiff shell is more capable of promoting the proliferation of MSCs and better supporting their residence in the body. Meanwhile, following the encapsulation of MSCs, single‐cell RNA sequencing analysis revealed a notable increase in the ratio of pluripotent MSCs to differentiated cells, thereby indicating the maintenance and restoration of cellular viability (Figure 3C).^[^ ^164^ ^]^ Moreover, the pertinent research has successfully developed an integrated microfluidic device capable of generating, extracting online, and dynamically culturing microspheres that encapsulate cells. The tumor‐endothelial coculture model established on this integrated platform demonstrates a significant expression of angiogenic proteins, thereby offering a more dependable analytical instrument for applications in tissue engineering and cancer therapy (Figure 3D).^[^ ^165^ ^]^ Based on the principle of unstable viscosity within fluids, achieving the spatial heterogeneous distribution of different types of cells within the same hydrogel microsphere is of great significance for vascularized tissue regeneration, stem cell therapy, and the construction of multicell spheres, especially for the study of different biophysical signals in terms of cell function and phenotype.^[^ ^166^ ^]^ However, the encapsulation of cells within microspheres results in a reduction of direct cellular interactions, which may have implications for research that necessitates intercellular signal transmission.^[^ ^167^ ^]^

When employed as cell culture carriers, HMs offer distinct advantages and limitations depending on whether cells are loaded internally or cultured on the surface. Internal cell entrapment is particularly effective for mimicking native cell–matrix interactions, whereas surface attachment is more suitable for achieving large‐scale cell expansion and efficient delivery.

### 4.3 Regenerative Medicine

HMs are extensively used in the repair and regeneration of diverse tissues, such as bone, cartilage, intervertebral disc, vascular, and nerve, owing to their injectable properties, substantial surface area, and the ability to regulate release characteristics (**Table** **3**). Meanwhile, HMs perform diverse functions by encapsulating a range of substances, including small‐molecule pharmaceuticals, metal ions, and cells.

**Table 3:** Comparison of HMs for regenerative medicine.

Tissues of regeneration	Materials	Methods	Mechanism	Reference
Bone	GelMA PLGA HA PLLA GelMA/HAMA	Emulsification Electrohydrodynamic Spraying Microfluidic Microfluidic Microfluidic	ALN inhibit osteoclast activity, Mg^2+^ promote osteogenic differentiation Good biocompatibility, promote osteogenesis efficiency Remodel gut–bone homeostasis, mitigate inflammation Sequential activation of osteogenic microenvironment Promote adhesion, proliferation, and osteogenic differentiation	[168] [169] [170] [171] [172]
Cartilage	HAMA ChsMA ChsMA Chs/SerMA GelMA	Microfluidic Microfluidic Microfluidic Microfluidic Microfluidic	Convert pressure into electrical stimulation, promote cartilage regeneration Inhibit chondrocyte senescence, regulate macrophage polarization, improve lubrication Improve the inflammatory microenvironment, mitigate oxidative stress Lubricate cartilage, ameliorate intra‐articular inflammation, promote cartilage repair Local recruitment of autologous stem cells, promote cartilage regeneration	[173] [174] [175] [60] [176]
IVDD	GelMA/ChsMA GelMA/Fucoidan GelMA GelMA–gelatin–vanillin GelMA	Microfluidic Microfluidic Microfluidic Microfluidic Microfluidic	Dual‐action antioxidant and cellular therapy Regulate the inflammatory microenvironment, improve ECM remodeling Bound to proinflammatory cytokines, biomimetic cell membrane‐coated Modulate oxygen tension in situ, to trigger endogenous cell recruitment and migration Promote inflammation inhibition and ECM regeneration	[48] [177] [178] [179] [180]
Vascular	Alginate/collagen Alginate Gelatin	Electro‐assisted bioprinting Electrohydrodynamic spraying emulsification	Facilitate HUVEC adhesion, proliferation, prevascularized tissue formation Limit the migration of the captured cells, provide privileged microenvironment Facilitate nerve infiltration and angiogenesis, guide the neurite outgrowth	[160] [181] [182]
Nerve	PLGA–GelMA GelMA Peptide‐based hydrogel GelMA PLGA	Microfluidic Electrohydrodynamic spraying Electrohydrodynamic spraying Microfluidic Emulsification	Mg2^+^ reduce inflammation, Zn^2+^ promote neural cell proliferation and regeneration Enhance NSC adhesion, proliferation, and differentiation Stimulate axon regeneration, promote the survival of NSCs and neuronal differentiation Reduce macrophage M2 polarization and inflammatory, promote nerve regeneration Regulate polarization of spinal microglial/macrophages, target release	[183] [184] [185] [186] [187]

**Figure 4:**
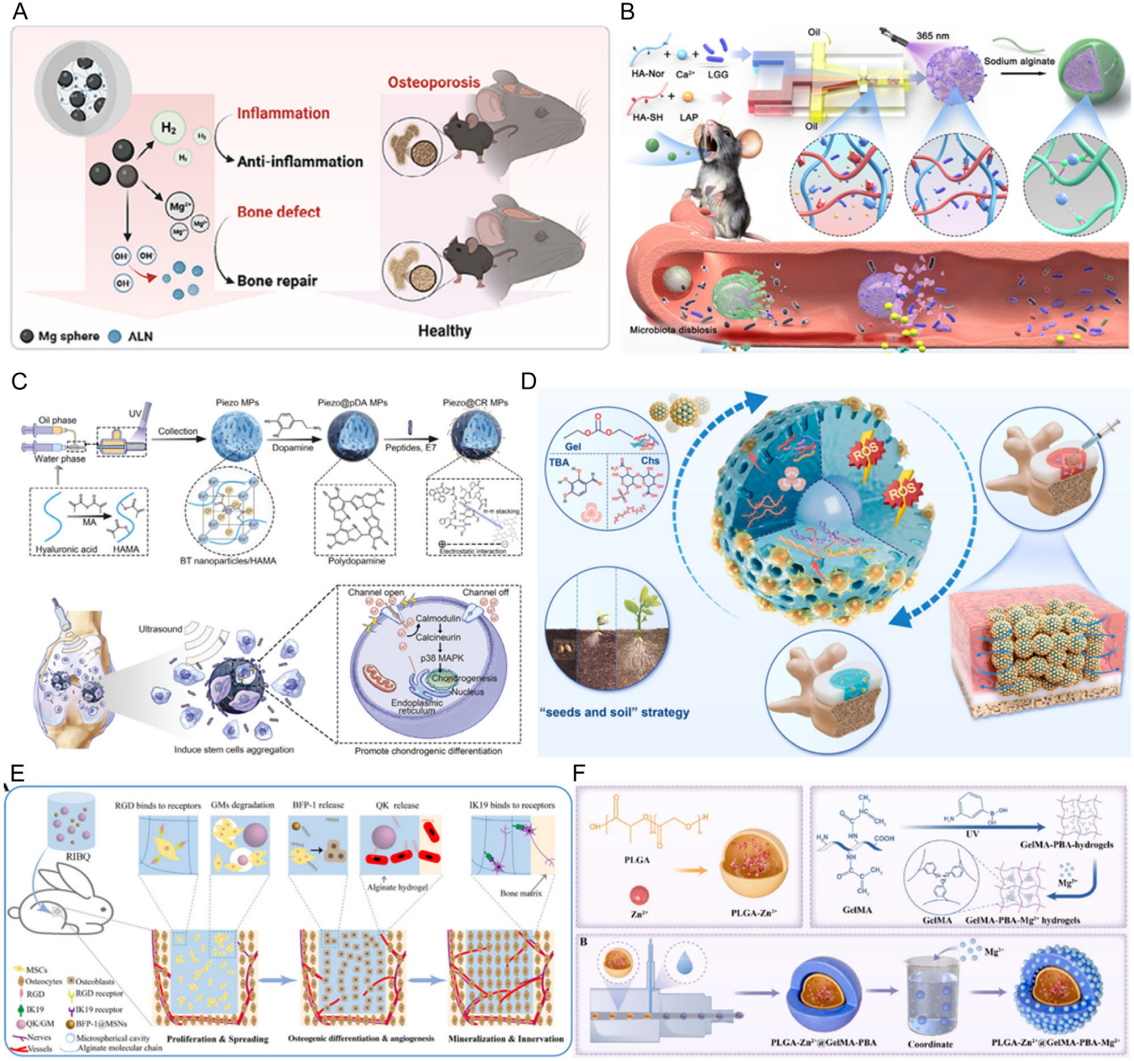
HMs for regenerative medicine. A) Schematic illustration of the use of multicore functional microspheres for the treatment of osteoporotic bone defects. Reproduced with permission.^[^ ^168^ ^]^ Copyright 2025, John Wiley & Sons. B) In situ reconstruction of gut–bone homeostasis with self‐replenishable metabolically augmented synbiotic microspheres for treating postmenopausal osteoporosis. Reproduced with permission.^[^ ^170^ ^]^ Copyright 2025, John Wiley & Sons. C) Force‐electric conversion HMs for cartilage injury treatments. Reproduced with permission.^[^ ^173^ ^]^ Copyright 2024, John Wiley & Sons. D) HMs demonstrate multifunctional efficacy by scavenging ROS, promoting NPC proliferation, and facilitating ECM remodeling, thereby contributing to NP repair. Reproduced with permission.^[^ ^48^ ^]^ Copyright 2025, Elsevier. E) Schematic diagram of the experimental design of neurovascularized bone repair system. Reproduced with permission.^[^ ^182^ ^]^ Copyright 2023, The Royal Society of Chemistry. F) Schematic illustration of the “core–shell” chimeric microspheres for repair of the complete transection‐induced spinal cord. Reproduced with permission.^[^ ^183^ ^]^ Copyright 2025, Elsevier.

In bone regeneration, HMs have been widely applied in bone tissue repair and regeneration. Nevertheless, conditions such as osteoporosis, along with bone abnormalities resulting from trauma, infection, and the aging process, can significantly impair the quality of life for affected individuals. The multicore functional HMs loaded with alendronate sodium and magnesium significantly improve the inflammatory microenvironment associated with osteoporotic bone defects through the synergistic effect of multiple mechanisms, which inhibit osteoclast activity and promote osteoblast differentiation (**Figure** **4**A).^[^ ^168^ ^]^ Furthermore, the integration of ultrasound‐activated injectable sodium alginate scaffolds with electrospun microspheres facilitated the regulated release of naringin in response to ultrasound stimulation, ensuring the safe and precise delivery of small molecule drugs and thereby enhancing bone regeneration capacity.^[^ ^169^ ^]^ In addition, the self‐supplementing metabolically enhanced synthetic microspheres loaded with hyaluronic acid and Lactobacillus rhamnosus ameliorate osteoporosis by regulating the intestinal microbiota, providing a theoretical framework for prospective clinical applications (Figure 4B).^[^ ^170^ ^]^ The composite peptide‐modified microfluidic microspheres, which incorporate stem cell‐homing peptides alongside BMP‐2 mimetic peptides, facilitate bone regeneration through the sequential activation of an osteogenic microenvironment.^[^ ^171^ ^]^ Meanwhile, the relevant research developed an injectable bone repair strategy by encapsulating stem cells in HMs for targeted delivery to injury sites. Photocrosslinked GelMA/HAMA microspheres, engineered via microfluidic technology to replicate an osteogenic microenvironment, were used to host BMSCs. Subsequently, these constructs were induced to form bone regeneration units, which ultimately facilitated both ectopic osteogenesis and the repair of tibial defects in a rabbit model.^[^ ^172^ ^]^

In cartilage regeneration, articular chondrocytes exhibit a low density and are devoid of nerves and blood vessels, which significantly restricts their capacity for self‐repair. Therefore, the regeneration of cartilage presents considerable challenges. However, the HMs have made some progress in cartilage regeneration. A force‐electric conversion hydrogel microsphere, which generates local electrical stimulation by activating nano‐piezoelectric particles with ultrasound, precisely promotes the migration of MSCs and facilitates cartilage differentiation through the recruitment of peptides by connecting cells and ultimately contributing to the repair of cartilage (Figure 4C).^[^ ^173^ ^]^ Moreover, nicotinamide adenine dinucleotide‐loaded lubricated HMs demonstrate efficacy in mitigating age‐related osteoarthritis through a multifaceted approach that includes the inhibition of chondrocyte senescence, the modulation of macrophage polarization, and the enhancement of joint lubrication.^[^ ^174^ ^]^ Furthermore, the injectable chondroitin sulfate microspheres incorporating gallic acid–magnesium metal‐organic framework exhibit significant therapeutic potential for osteoarthritis. They effectively mitigate inflammation and cartilage degeneration, thereby delaying disease progression through joint microenvironment modulation and enhanced cartilage repair.^[^ ^175^ ^]^ Moreover, the bionic bearing‐inspired lubrication microsphere with immunomodulatory function uses chondroitin sulfate and sericin methacryloyl as the backbone and combines folic acid‐targeted liposomes to achieve a “lubrication–anti‐inflammation–repair” trinity treatment for osteoarthritis.^[^ ^60^ ^]^ A TGF‐β‐affinity peptide‐modified, antibody‐mediated GelMA‐based hydrogel microsphere is designed to continuously recruit endogenous BMSCs through intra‐articular injection for precise cartilage regeneration, precisely targeting and repairing scattered cartilage damage, and is used for nonsurgical treatment of advanced osteoarthritis.^[^ ^176^ ^]^

In intervertebral disc regeneration, IVDD is a consequence of the natural aging process, while the regeneration of intervertebral discs necessitates a dual approach that addresses both tissue repair and functional restoration. A recent study showed that seeds‐and‐soil‐inspired HMs, promoting the survival, proliferation, and functional repair of NPCs, provide a new paradigm for the combination of antioxidant biomaterials and cell therapy in the treatment of IVDD (Figure 4D).^[^ ^48^ ^]^ Moreover, the research team has successfully created a Brachyury‐activated fucoidan hydrogel microsphere system, which offers an innovative approach for the treatment of IVDD. This system effectively modulates the inflammatory microenvironment and facilitates the regeneration of the ECM.^[^ ^177^ ^]^ Additionally, the immune‐defensive microspheres, in combination with neutrophil cell membranes and transforming growth factor‐β, reshape the ECM of intervertebral discs through inflammatory regulation, thereby providing a potential new approach for effectively coordinating inflammation and regeneration in IVDD treatment.^[^ ^178^ ^]^ Furthermore, relevant researchers have developed hypoxia‐induced interpenetrating polymer network HMs that are encapsulated with exosomes derived from neural stem cells. These microspheres facilitate the recruitment and differentiation of endogenous stem cells, activate the PI3K/AKT/HIF‐1α signaling pathway, and enhance the differentiation of NPCs as well as the synthesis of the ECM.^[^ ^179^ ^]^ Another study revealed that injectable HMs loaded with BMSCs and synthetic modified peptides provide a novel and effective strategy for the treatment of intervertebral discs by regulating the inflammatory microenvironment, promoting nucleus pulposus differentiation and ECM regeneration.^[^ ^180^ ^]^ In summary, HMs contribute significantly to the regeneration of intervertebral discs by enhancing ECM synthesis, modulating inflammatory responses, facilitating the delivery of bioactive molecules, improving the microenvironment, and promoting cell survival and differentiation.

In vascular regeneration, HMs can sustainably and minimally invasively deliver growth factors, essential trace elements, and other molecules to repair tissues through the regulation of various cellular processes, including cell attachment, proliferation, migration, differentiation, and interactions between cells and between cells and materials. Electrically assisted bioprinted microspheres composed of alginates and collagen, which are infused with collagen fibers and HUVECs, enhance the adhesion and proliferation of HUVECs and facilitate angiogenesis and offer potential approaches for the regeneration of vascularized tissues and the treatment of ischemia.^[^ ^160^ ^]^ Furthermore, HMs containing VEGF‐overexpressing HEK293T cells have been shown to significantly enhance angiogenesis in a mouse model of hind limb ischemia.^[^ ^181^ ^]^ A neurovascular HM composite material has been developed to promote nerve growth and angiogenesis by different peptides in the context of bone defects, reproduce the bone tissue microenvironment, and improve the repair effect of bone defects (Figure 4E).^[^ ^182^ ^]^ In summary, HMs demonstrate considerable promise for vascular regeneration. By modulating key cellular activities and establishing a favorable microenvironment, they offer a substantial therapeutic strategy to enhance neovascularization.

In nerve regeneration, HMs, emerging as a novel category of biomaterials, exhibit significant potential for application in the treatment of spinal cord injuries. By employing mechanisms that include the suppression of inflammatory responses and the facilitation of neuronal regeneration, these microspheres offer innovative therapeutic approaches for the repair of spinal cord injuries. The research team developed a core–shell HMs doped with magnesium and zinc, which sequentially releases metal ions to create a conducive microenvironment for nerve regeneration. This innovative design integrates the advantages of both materials, providing immediate mechanical support at the site of spinal cord injury while progressively modulating the pathological microenvironment through staged ion release (Figure 4F).^[^ ^183^ ^]^ In addition, the laminin‐modified porous GelMA microspheres provide a biomimetic 3D microenvironment for NSCs, which markedly improves both the adhesion rate and proliferative activity of these cells and offers a new and effective carrier for the cell transplantation therapy of SCI.^[^ ^184^ ^]^ Recent investigations have developed a platelet‐derived growth factor analog peptide HMs (PDGF‐MPHMs), which was cotransplanted alongside neural stem cells into the injured spinal cord of rat models. This approach effectively enhanced the recovery of motor function in rats with spinal cord injuries by mitigating apoptosis in the affected region, reducing the infiltration of M1‐type macrophages, and promoting axonal regeneration, remyelination, and angiogenesis.^[^ ^185^ ^]^ Furthermore, core–shell HMs, in conjunction with the sustained release of cerium oxide nanoparticles and the ECM derived from spinal white matter, mitigate early inflammatory responses during the acute phase by promoting M2 polarization of macrophages and microglia while decreasing the infiltration of inflammatory cells. This intervention ultimately contributes to enhanced functional recovery following spinal cord injury.^[^ ^186^ ^]^ The injectable and sustained‐release PLGA microspheres, which are incorporated with melatonin and platelet membrane, have been shown to enhance the repair of spinal cord injuries in rats by modulating the polarization of spinal microglia and macrophages.^[^ ^187^ ^]^ Subsequent investigations should focus on refining the material characteristics of HMs, improving their biocompatibility and therapeutic efficacy, and facilitating their transition from preclinical animal studies to clinical applications.

Moreover, there are some HMs that function in more than one type of regeneration. Implantable multifunctional HMs are designed to modify and enhance the microenvironment, thereby facilitating the processes of vascular and bone regeneration. The CaO_2_@SiO_2_@PDA nanoparticles demonstrate effective binding to porous GelMA microspheres via amide bonds, which imparts sustained oxygen release capabilities and enhances the ROS scavenging potential.^[^ ^188^ ^]^ Furthermore, the biosignal‐integrated microfluidic HMs system, which incorporates signal peptides, synergistically combines osteogenic and angiogenic signal peptides, promoting bone tissue regeneration at bone defects by inducing osteogenic differentiation and neovascularization.^[^ ^189^ ^]^ In addition, inspired by the lotus structure, nano‐microscale HMs were developed to efficiently encapsulate periodontal ligament stem cells pretransfected with polydopamine/berberine nanoparticles and bone morphogenetic protein 9. These microspheres demonstrated robust antibacterial and anti‐inflammatory effects, alongside a pronounced ability to promote periodontal tissue regeneration, thereby offering a novel therapeutic strategy for periodontitis.^[^ ^190^ ^]^

HMs exhibit significant promise in the field of regenerative medicine; however, they encounter several challenges. Notably, the majority of HMs are derived from a limited number of material systems, including gelatin, HA, alginate, and PEG. Consequently, numerous biomaterials possessing biomimetic properties and biological functionalities have yet to be utilized in the fabrication of HMs. This limitation primarily arises from their insufficient crosslinking capabilities or the absence of rapid crosslinking techniques.

### 4.4 Wound Healing

**Figure 5:**
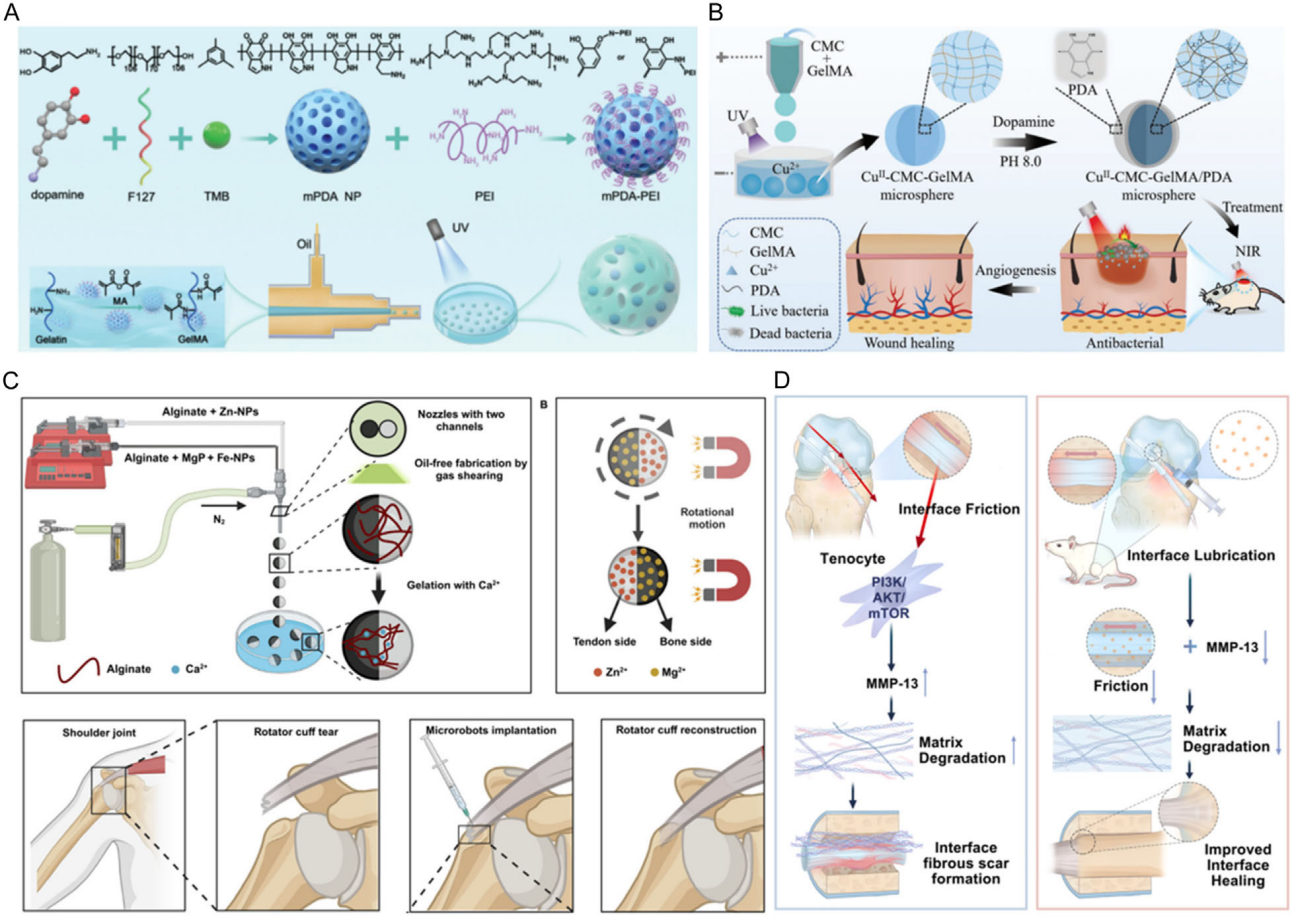
HMs for wound healing. A) Synthesis process of neutrophil extracellular traps scavenger microcage mPDA‐PEI@GelMA and its application in treating wounds in diabetic mice. Reproduced with permission.^[^ ^191^ ^]^ Copyright 2024, John Wiley & Sons. B) Illustration of fabrication and application of the CuII‐CMC‐GelMA/PDA microspheres. Reproduced with permission.^[^ ^195^ ^]^ Copyright 2023, John Wiley & Sons. C) Fabrication and application of magnetic Janus hydrogel microrobots in rotator cuff tear reconstruction surgery. Reproduced with permission.^[^ ^196^ ^]^ Copyright 2025, Springer Nature. D) Illustration of the impact and mechanism of interface friction and lubrication on interface healing via lubricating microspheres. Reproduced with permission.^[^ ^197^ ^]^ Copyright 2025, Elsevier.

Wound healing represents a multifaceted and intricately regulated biological process through which the skin and other tissues in the human body undergo self‐repair to regain their structural integrity and functionality following an injury. This dynamic process unfolds in multiple stages and necessitates the coordinated interaction of diverse cellular components, cytokines, growth factors, and the ECM. HMs exhibit a range of functionalities pertinent to wound healing, such as the regulation of the microenvironment, antibacterial and anti‐inflammatory effects, facilitation of cell proliferation, and controlled drug release. The innovative biobased HMs mitigate the inflammatory response associated with chronic diabetic wounds and promote healing by degrading the extracellular traps produced by neutrophils (**Figure** **5**A).^[^ ^191^ ^]^ Moreover, the incorporation of living bacterial (Lactobacillus) into photosensitive HMs facilitates the formation of an in situ gel at the wound site. Concurrently, this approach enhances the healing process of infected wounds by leveraging the antibacterial compounds secreted by the probiotics. This mechanism not only serves to shield bacteria from immune system responses but also inhibits their dissemination into the surrounding environment, thereby mitigating potential risks.^[^ ^192^ ^]^ The healing of cutaneous wounds comprises four phases, hemostasis, inflammation, proliferation, and remodeling, in which precise pH modulation plays a critical role. The microgel ensembles sustain an acidic environment during the inflammatory and hemostatic phases to suppress bacterial growth; conversely, they provide an optimal alkaline milieu during proliferation and remodeling to promote fibroblast proliferation and migration, thus overall accelerating wound healing.^[^ ^193^ ^]^ Relevant research has demonstrated that a charge‐driven self‐assembled hydrogel microsphere scaffold, which is infused with black phosphorus and basic fibroblast growth factor, when utilized in conjunction with short‐term photothermal treatment and sustained drug release, effectively mitigates inflammatory responses, enhances angiogenesis and tissue remodeling, and markedly expedites the wound healing process.^[^ ^194^ ^]^ Moreover, the composite microspheres with dual antibacterial mechanisms and bioadhesion properties continuously release copper ions, demonstrating long‐term antibacterial properties and playing a significant role in angiogenesis related to wound healing. Meanwhile, the microspheres are coated with polydopamine via a self‐polymerization process, which enhances their adhesion to the wound surface and further amplifies their antibacterial properties through the conversion of photothermal energy (Figure 5B).^[^ ^195^ ^]^ In summary, HMs enhance the healing process of cutaneous wounds and offer novel perspectives on strategies for wound healing.

However, HMs also play a certain role in some other healing processes. Interestingly, magnetic Janus alginate microspheres featuring a dual‐compartment architecture have been developed, with one compartment containing Mg^2+^ and the other containing Zn^2+^. The presence of Mg^2+^ has been shown to enhance the proliferation, migration, and osteogenic differentiation of BMSCs, while Zn^2+^ plays a crucial role in collagen remodeling and tendon regeneration. Collectively, these properties contribute to the effective healing of the shoulder cuff‐bone interface (Figure 5C).^[^ ^196^ ^]^ Another study revealed that the application of lubricating HMs integrates lubrication with the inhibition of matrix degradation, thereby facilitating interfacial healing through the downregulation of the PI3K/AKT/mTOR/MMP‐13 signaling pathway (Figure 5D).^[^ ^197^ ^]^

### 4.5 Tumor Immunity

**Figure 6:**
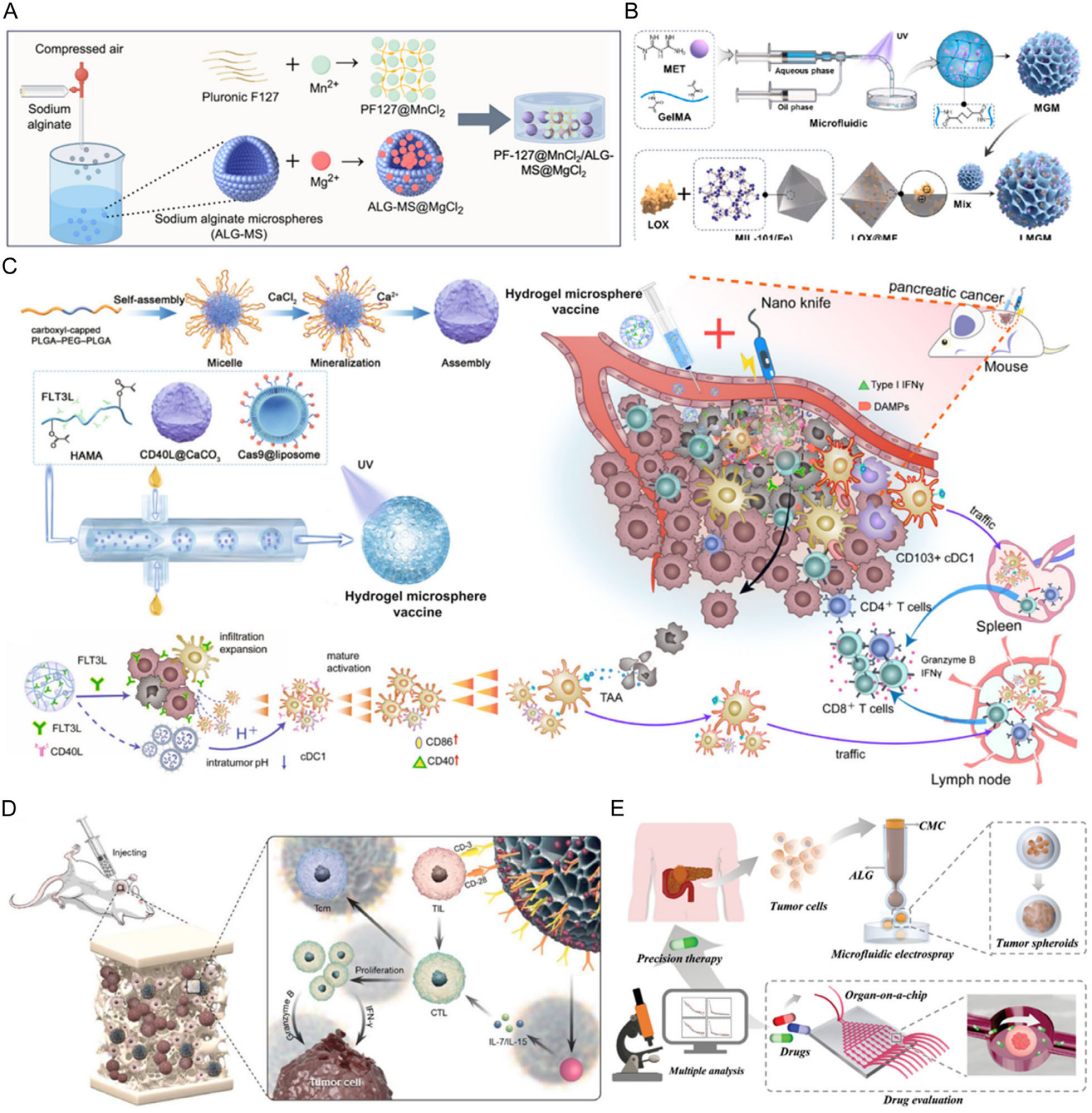
HMs for tumor immunity. A) Synthesis of the PF‐127@MnCl_2_/ALG‐MS@MgCl_2_ composite hydrogel. Reproduced with permission.^[^ ^200^ ^]^ Copyright 2024, Elsevier. B) Fabrication of antitumor and antimetastasis of LOX@MF and MET dual‐loaded microspheres. Reproduced with permission.^[^ ^27^ ^]^ Copyright 2025, Elsevier. C) The cDC1‐activated hydrogel microsphere vaccine was used as a general immune amplifier to amplify the cDC1/CD8+ T‐cell antitumor axis after ablation therapy. Reproduced with permission.^[^ ^202^ ^]^ Copyright 2023, Springer Nature. D) Microsphere‐integrated training court was injected orthotopically into osteosarcoma tumor‐bearing mice to simultaneously activate anergy of TIL‐Ts and increase the formation of CTLs and memory T cells, consequently killing tumor cells and promoting tumor regression. Reproduced with permission.^[^ ^203^ ^]^ Copyright 2024, Elsevier. E) Diagram illustrating the peripheral vasculature of pancreatic tumors alongside a microfluidic chip embedded with hydrogel microcapsules designed for drug assessment. Reproduced with permission.^[^ ^207^ ^]^ Copyright 2023, John Wiley & Sons.

As advanced biomaterial carriers, HMs hold great promise in tumor immunotherapy. Their unique properties help overcome major constraints of conventional methods, with applications spanning targeted drug delivery, TME simulation, and immunotherapy, positioning them as a comprehensive and transformative platform in oncology research. The immunoamplification HMs loaded with photosensitizer cypate and STING agonist MSA‐2 activate the STING signaling pathway to enhance immunity, reverse immunosuppression, and transform cold tumors into hot tumors.^[^ ^198^ ^]^ Moreover, the Ni–alginate HMs demonstrate a notable capacity for loading interleukin‐2 (IL‐2) and facilitating its sustained release postadministration within tumor environments, significantly increasing tumor infiltration of T lymphocytes and effectively inhibiting tumor growth, especially when used in combination with immune checkpoint inhibitors.^[^ ^199^ ^]^ Furthermore, relevant researchers have developed temperature‐sensitive HMs that control the rapid release of Mn^2+^ and the gradual and continuous release of Mg^2+^, creating a cytotoxic immune microenvironment conducive to CD8 T cells and natural killer cells, thereby enhancing the efficacy of immune checkpoint blockade therapy (**Figure** **6**A).^[^ ^200^ ^]^ Another study revealed that an injectable thermosensitive microsphere‐hydrogel composite system was used for the localized codelivery of the targeted drug sorafenib and the immunomodulatory cytokine IL‐12, achieving combined treatment of hepatocellular carcinoma by reshaping the tumor immune microenvironment and significantly enhancing the antitumor effect.^[^ ^201^ ^]^ Furthermore, locally administered glycolysis‐modulating HMs utilized a metabolic‐trapping strategy based on lactate oxidase and metformin, which demonstrated potent inhibition of tumor proliferation and angiogenesis. In conclusion, this study offers a novel strategy to overcome the challenge of metabolic heterogeneity, thereby broadening the therapeutic scope of metabolic interventions in cancer treatment (Figure 6B).^[^ ^27^ ^]^ Injectable HM immunotherapy not only simplifies surgical procedures and alternative treatment modalities but also markedly enhances the effectiveness of immunotherapeutic interventions.

In tumor vaccine and construction of tumor model, relevant research has developed an injectable hydrogel microsphere vaccine that can stimulate the cDC1‐mediated antigen cross‐presentation cascade. The integration of this hydrogel vaccine with ablation therapy has been shown to significantly enhance the proliferation of cDC1/CD8^+^ T cell‐mediated antitumor immunity, thereby impeding the advancement of pancreatic cancer and the proliferation of distant metastases (Figure 6C).^[^ ^202^ ^]^ Interestingly, the HM‐integrated training field was established within the tumor via localized injection, facilitating dual signaling mechanisms that activate tumor‐infiltrating T lymphocytes. This system enables the sustained release of IL‐7 and IL‐15, which are critical for preserving the viability of tumor‐infiltrating T lymphocytes and enhancing the development and persistence of cytotoxic T lymphocytes and memory T cells (Figure 6D).^[^ ^203^ ^]^ The construction of tumor models is essential for identifying novel drug targets. More importantly, the honeycomb‐structured HMs have the potential to replicate the physiological microenvironment of tumors, preserving both cell–cell and cell–ECM interactions. Consequently, they offer a viable and efficient approach for investigating tumor pathogenesis and conducting drug screening research.^[^ ^204^ ^]^

The interaction between tumor cells and stromal cells plays a critical role in tumor metastasis. Developing a reliable model to study this cellular crosstalk is thus crucial for elucidating metastatic mechanisms and informing therapeutic strategies. The 3D tumor matrix coculture microsphere model enables direct observation of reciprocal activation between tumor cells and fibroblasts, while maintaining the integrity of key metabolic pathways such as the arginine–ornithine–citrulline cycle.^[^ ^205^ ^]^ A microtumor was developed within a singular HMs, with meticulous regulation of its dimensions, cellular composition, and morphology, thereby establishing a standardized assessment model in vitro.^[^ ^206^ ^]^ Additionally, an innovative category of microfluidic HMs has been developed, capable of encapsulating primary human pancreatic cancer cells to create 3D biomimetic tumor spheres for providing a reliable and accurate drug evaluation platform for the clinical treatment of pancreatic cancer (Figure 6E).^[^ ^207^ ^]^

HMs serve as effective delivery vehicles for tumor immunity, offering a robust technical framework for the advancement of novel tumor immunity that are both efficient and safe. Their exceptional capabilities in codelivery, sustained release, targeting delivery, and the construction of immune regulatory microenvironments, along with their potential for multifunctional integration, position them as promising solutions to the challenges currently faced by existing tumor immunity. The efficacy of antitumor immunotherapy is limited by persistent hurdles, including poor delivery efficiency, inadequate immunogenicity, and the inability to reverse immunosuppressive tolerance. The continued integration of materials science, nanotechnology, and immunology, together with advances in fabrication techniques, positions HMs as promising platforms to increasingly contribute to cancer immunotherapy and advance toward clinical application.

### 4.6 Other Biomedical Applications

HMs, with their adjustable structural characteristics, excellent biocompatibility, and versatile fabrication methods, have demonstrated significant potential for diverse biomedical applications, including organoid technologies. Organ‐on‐a‐chip technology constitutes an in vitro platform that employs microfluidic systems to replicate the microphysiological conditions characteristic of human organs. Specifically, HMs possess the capability to replicate the architecture and functionality of ECM, thereby offering 3D environment for cell growth. Furthermore, their chemical composition and physical characteristics can be precisely engineered to emulate diverse tissue‐specific microenvironments.^[^ ^208^ ^]^ In addition, HMs play a pivotal role in mitigating the significant issue of batch‐to‐batch variability in organoid production by offering a uniform and standardized culture environment.^[^ ^209^ ^]^ More importantly, HM‐based organoid cultures are well suited for high‐throughput drug screening and toxicity assessment owing to their discrete structure and ease of manipulation.^[^ ^74^, ^210^ ^]^ For example, relevant researchers have successfully developed conformal bone organoid units by fabricating silk fibroin HMs endowed with sustained oxygen release and immunomodulatory properties systematically investigated the functions and underlying mechanisms of bone regeneration.^[^ ^211^ ^]^ Moreover, through the uniform encapsulation of cells obtained from the patient's TME and lung cancer organoids within HMs, it is feasible to accurately handle clinical trace samples and swiftly produce lung cancer aggregates exhibiting consistent size and cellular composition across batches. This approach enables the reconstruction of the functional heterogeneity of cancer‐associated fibroblasts and more effectively captures the influence of the TME on drug response in comparison to conventional cancer organoids.^[^ ^212^ ^]^ In summary, HMs, as a significant biomedical resource, exhibit substantial potential for applications in organoid technologies.

## 5 Clinical Translation and Trials

The microsphere drug delivery system facilitates sustained and controlled drug release, thereby improving drug stability. This technology holds significant potential for clinical applications and has emerged as a leading and highly active area in the development of novel pharmaceutical formulations. Numerous HMs products, originating from natural materials, synthetic materials, or their composites, have received approval from the US Food and Drug Administration (FDA), such as Embosphere, DC Bead, and Ellansé. Embosphere represents a sustainable approach to TACE therapy, capable of permanently occluding peripheral blood vessels while eliciting only mild inflammatory reactions, thereby facilitating the treatment of tumors and fibroids.^[^ ^213^ ^]^ DC Bead microspheres function as embolic agents that not only occlude blood vessels but also act as delivery vehicles for localized chemotherapeutic agents. These microspheres enable the targeted administration of high concentrations of chemotherapy drugs directly to tumor sites, facilitating sustained drug release. This localized delivery system substantially minimizes systemic toxicity and adverse side effects in unresectable hepatocellular carcinoma.^[^ ^214^ ^]^ Ellansé consists of 30% synthetic PCL microspheres suspended in a 70% aqueous CMC carrier, which have uniform size and smooth and spherical format. It serves as a durable dermal filler, designed to enhance volume while promoting endogenous collagen regeneration.^[^ ^215^ ^]^

Notwithstanding, certain HMs are presently being investigated in clinical research, such as OptiSphere (NCT04951479) and CartiLife (NCT05051332). The OptiSphere is crosslinked using glutaraldehyde, a process that enhances the mechanical strength of the spheres and facilitates controlled degradation following implantation. The spherical morphology ensures smooth embolic delivery as well as uniform and predictable distribution. In the present study, the OptiSphere will be utilized for geniculate artery embolization, a minimally invasive procedure traditionally employed in the treatment of patients experiencing recurrent knee hemarthrosis.^[^ ^216^ ^]^ Furthermore, the CartiLife HMs in phase III clinical trials consists of fibrin microspheres designed for the treatment of articular cartilage defects and degeneration by intraarticular injection.^[^ ^217^ ^]^ More importantly, the conjugation of the IL‐15 agonist or receptor‐linker IL‐15 with HMs substantially prolonged the half‐life of IL‐15, improved its pharmacodynamic properties, and conferred advantageous antitumor and antimetastatic effects.^[^ ^218^ ^]^Meanwhile, the long‐acting intratumoral HMs loaded with SN‐38, a topoisomerase inhibitor, in combination with a PARP inhibitor, provide a generic approach for safe use of drug and immunotherapy combinations with overlapping toxicities.^[^ ^219^ ^]^

Clinical HMs in the future will be more intelligent and functional. Firstly, stimuli‐responsive HMs, which react to factors such as pH, temperature, and enzymatic activity, have been developed to enable more controlled and targeted drug delivery.^[^ ^220^ ^]^ More importantly, HMs have the capability to encapsulate contrast agents, thereby facilitating the combined application of diagnostic imaging and therapeutic intervention. Despite the numerous challenges encountered in the clinical translation of microsphere‐based drug delivery systems, such as the precise regulation of drug release kinetics and the absence of standardized manufacturing equipment, the advancement and implementation of these systems can be expedited through interdisciplinary collaboration among pharmacy, materials science, and related disciplines. Such cooperative efforts are essential to address the obstacles associated with the scale‐up of microsphere production and to continuously refine the fabrication processes of drug delivery microspheres.

## 6 Future and Shortcomings

Despite the promising applications of HMs in the biomedical sector, they continue to encounter several challenges and limitations. 1) First, the majority of HMs are derived from a limited number of material systems. Future advancements should focus on the development of novel materials that possess biomimetic characteristics and biological functionalities to address the diverse requirements of various biomedical applications. Furthermore, HMs can be synthesized utilizing composite materials that incorporate both natural polymers and synthetic substances, thereby improving their biocompatibility and functional properties.^[^ ^221^ ^]^ The biphase sustained‐release composite material, which incorporates submucosal hydrogel derived from the small intestine and sodium alginate microspheres, demonstrates an effective capacity to attract tendon‐derived stem cells and facilitate their differentiation into tendon‐forming cells through a controlled, staged release of SDF‐1α and BMP‐12.^[^ ^222^ ^]^ 2) Second, future investigations into HMs are anticipated to prioritize multifunctionality and intelligent design. For example, HMs fabricated via microfluidic technology enable precise regulation of drug and cell delivery, thereby improving the accuracy and efficacy of therapeutic interventions. 3) Furthermore, advancements in 3D printing technology are expected to enhance the application of HMs in the realms of personalized medicine and tissue engineering. 4) HMs can function as primary scaffolding materials that facilitate cellular self‐organization, leading to the formation of more structurally complex organoids that more accurately recapitulate human organ architecture. These organoids are subsequently utilized for applications such as disease modeling and pharmaceutical screening. 5) HMs should be developed to attain multifunctional integration, incorporating capabilities such as drug delivery, cell delivery, and biosensing within a single platform. For example, these microspheres can be engineered to concurrently encapsulate pharmaceuticals, growth factors, and cells, thereby facilitating synergistic therapeutic effects.^[^ ^206^ ^]^ Furthermore, the antibacterial efficacy of HMs can be improved through various functionalization strategies, including the incorporation of antimicrobial peptides and photothermal nanomaterials. 6) However, the integration of nanomaterials with HMs, although providing multifunctional capabilities, introduces novel challenges primarily attributable to the pronounced disparities in the physical, chemical, and biological characteristics of nanomaterials and hydrogel matrices, alongside the intricate nature of their interfacial interactions.^[^ ^223^ ^]^ Consequently, these challenges may be addressed by employing surface modification techniques for nanomaterials, optimizing synthesis protocols, and implementing molecular design strategies. 7) In terms of microenvironment regulation, HMs must enhance their microenvironment to more accurately replicate the properties of the ECM. For instance, by adjusting the mechanical characteristics, pore structure and surface chemical properties of hydrogels, a more suitable growth environment for cells can be provided. In summary, the future advancement of HMs is anticipated to transcend mere enhancements of current materials, progressing toward a novel phase characterized by the balanced emphasis on intelligent design, technological integration, and clinical translation. Through the incorporation of artificial intelligence, advanced manufacturing techniques, and biological understanding, HMs can be transformed from multifunctional platforms into next‐generation biomedical tools capable of dynamic sensing, precise responsiveness, and effective life repair. This evolution holds significant potential to pave new avenues in the field of precision medicine.

Despite significant advancements in laboratory research on HMs, the translation to clinical practice continues to face numerous obstacles, necessitating the resolution of several challenges. 1) It is imperative to conduct extensive animal studies and clinical trials to assess their safety and efficacy comprehensively. 2) In addition, challenges related to the mechanical strength, biocompatibility, and degradation rate of HMs must be resolved to satisfy the requirements for clinical use. 3) In the forthcoming developments of HMs, there is a projected emphasis on the integration of intelligence and personalization. For example, the implementation of responsive design can facilitate intelligent drug release mechanisms, thereby improving the therapeutic efficacy of pharmaceuticals. 4) In particular, an interdisciplinary methodology that integrates materials science, computer science, and biology has the potential to substantially accelerate the research and development timeline, decrease associated costs, and enable the discovery of novel materials exhibiting exceptional performance and intricate functionalities. For example, through the application of artificial intelligence to develop a relational model linking process parameters with the compressive modulus and pore size distribution of microspheres, a microsphere scaffold capable of replicating the mechanical properties of natural tissues has been designed. 5) Furthermore, through personalized design approaches, the dimensions, morphology, and functionalities of HMs can be tailored to meet the individual requirements of patients, thereby advancing the field of precision medicine. 6) However, as a complex combination product, HMs undergo a highly intricate regulatory approval process that necessitates the simultaneous fulfillment of multiple safety and efficacy criteria, thereby substantially extending the duration and increasing the cost of clinical trials.

In conclusion, the successful clinical translation of HMs should be guided by three fundamental strategies. First of all, the advancement of artificial intelligence should be prioritized to enable precise functional modulation through AI‐driven and responsive design approaches. In addition, the personalization of applications must be achieved by tailoring the properties of HMs to meet the specific anatomical and physiological requirements of individual patients. More importantly, it is essential to establish an integrated translational framework, which involves interdisciplinary collaboration and proactive regulatory strategies to systematically facilitate the entire process from laboratory research to clinical implementation. It is through such comprehensive and coordinated efforts that HMs truly bridge the gap from “promising” to “applicable.”

## 7 Conclusion

The application of HMs in drug delivery, cell culture, regenerative medicine, wound healing, and tumor immunity holds significant potential for advancing biomedical therapies. Their multifunctionality, adaptability, and responsiveness position them as a crucial area of investigation within the biomedical sector. To fully realize their clinical potential, interdisciplinary collaboration among biomaterials scientists, biomedical engineers, and clinicians will be essential. Such partnerships can accelerate the development of safe, scalable, and cost‐effective hydrogel microsphere‐based therapies. Moreover, addressing regulatory challenges and manufacturing hurdles remains critical to facilitate their successful translation from the laboratory to clinical practice. With continued innovation and cooperation, HMs are poised to play an increasingly vital role in improving human health in the near future.

## Conflict of Interest

The authors declare no conflict of interest.
